# Vertical DNA Nanostructure Arrays: Facilitating Functionalization on Macro-Scale Surfaces

**DOI:** 10.1021/acsnano.5c03100

**Published:** 2025-04-09

**Authors:** Hyeonjun Kwon, Jihoon Shin, Siqi Sun, Rong Zhu, Sarah Stainer, Peter Hinterdorfer, Sang-Joon Cho, Dong-Hwan Kim, Yoo Jin Oh

**Affiliations:** † School of Chemical Engineering, 35017 Sungkyunkwan University, Suwon 16419, Republic of Korea; ‡ Department of Applied Experimental Biophysics, Institute of Biophysics, 27266 Johannes Kepler University Linz, Gruberstrasse 40, A-4020 Linz, Austria; § 609103 Park Systems, Corp., KANC 15F, Gwanggyo-ro 109, Suwon 16229, Republic of Korea

**Keywords:** DNA nanotechnology, AFM, aptamer, macro-scale, supporting substrate, double-crossover tile

## Abstract

The capability for varied functionalization and precise control at the nanoscale are significant advantages of DNA nanostructures. In the assembly of DNA nanostructure, the surface-assisted growth method utilizing double-crossover (DX) tile structures facilitates nucleation at relatively low concentrations on the surface based on electrostatic interactions, thereby enabling crystal growth over large areas. However, in surface-assisted growth, the geometrical hindrance of vertical structures on the DX tile structure surface makes it challenging to conjugate DNA nanostructures into fabricated surfaces. Here, the surface-assisted growth method was employed to extend the DX tile growth for forming vertical structure arrays on the substrate, providing attachment sites for functionalization on uniformly covered substrates at the macroscopic scale. Additionally, the spacing of the vertical structure arrays was demonstrated to be controllable through the strategic design of the repeating unit tiles that construct the DX crystals.

## Graphical abstract

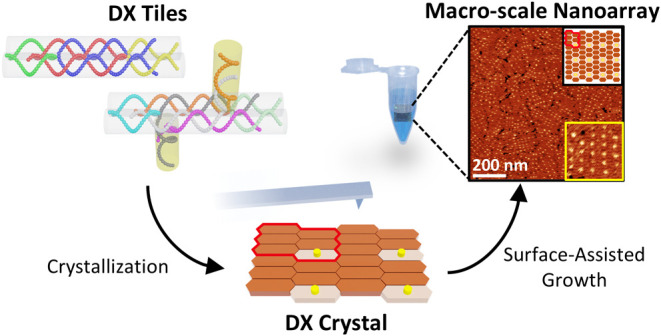

In crystallography, crystal growth refers to the process by which a substance forms a regular three-dimensional (3D) crystal structure through various techniques and methods.^1–4^ This process involves nucleation followed by growth, in which unit cells repeatedly bond to form a crystal^5,6^ and includes notable methods such as Chemical Vapor Deposition (CVD)^7,8^ and Molecular Beam Epitaxy (MBE).^9,10^ In crystal growth, a supporting substrate plays a crucial role in achieving the designed structures with high quality. The substrate interacts with the surface to promote heteroepitaxy,^11,12^ enabling the growth of high-purity,^13–16^ low-defect crystals over large areas.^17,18^ Using a supporting substrate is essential for manufacturing applications that require uniform and large-area crystals, such as in the production of semiconductor wafers^19,20^ and solar panels.^21^

In addition to these advances, one of the most promising crystal growth methods of the past decade is the use of DNA tile structures. DNA tiles, synthetic DNA molecules designed to self-assemble in a predetermined highly ordered pattern, have been a pioneering new approach.^22,23^ Their unique ability to form precise, programmable, and highly ordered features has led to advances in the development of complex nanodevices,^24,25^ scaffolds for studying biomolecular interactions,^26^ carriers for targeted drug delivery,^27,28^ etc.^29–31^ DNA tile structures utilize the unique properties of DNA base pairs to create complex nanostructures with a high degree of precision and predictability.^32^ Unlike traditional crystallography methods, which require complex procedures, DNA tiles can self-assemble into any desired pattern under the proper parameters.^33–35^ This unique self-assembly property implies the crystallization process and allows for achieving customizable crystal structures. By designing DNA sequences that bind specifically to complementary strands, tiles that uniformly incorporate functional groups or molecular components can be created,^36^ resulting in a uniform and predictable functional environment within the crystal. These features are critical for many applications, such as the development of materials with consistent mechanical,^37,38^ electrical,^39^ and optical properties.^40^ Especially, in the case of DNA tiles, a process akin to the crystal growth of other materials occurs, where repetitive tiles bond through nucleation and growth to form a DNA crystal.^41^ The surface-assisted growth method, similar to crystal growth involving a supporting substrate, promotes nucleation at relatively low concentrations on the surface based on electrostatic interactions, enabling large-area crystal growth.^42,43^

While the pursuit of large-scale crystal growth and functionalization has traditionally guided DNA tile-based structures, DNA origami structures likewise share this goal.^44^ DNA nanostructures have attracted significant attention for their potential in large-scale production, and continued research is expanding this potential to macro-scale applications. DNA origami, which leverage the binding of thousands of DNA base pairs, offer remarkable versatility for fabricating 1-, 2-, and 3-dimensional structures, with extensive possibilities for surface functionalization due to its high degree of design freedom.^45–52^

Early studies successfully produced larger origami structures by designing complex connections. So far published strategies include linking origami units with dozens of connection points to assemble multiple origami structures;^53,54^ using geometrical complementarity (e.g., blunt-end interactions);^55,56^ or enhancing the thermal stability of connections through chemical cross-linking.^57^ These approaches have paved the way for further applications aimed for macro-scale fabrication. In addition, surfaces with weak-connected DNA origami stacking patterns have been created that fully covered macro-scale surfaces, underscoring the broad potential of combining DNA nanostructures at surface interfaces with programmable assembly.^58^

Yet, while the use of supporting substrates for crystal growth is a crucial process in modern manufacturing, DNA surface-assisted growth has not received the same level of attention due to two key differences. First, although DNA has insulating properties^59,60^ and offers nanoscale controllability,^61,62^ it lacks the advantages of physical properties required for practical devices compared to materials like graphene^63–65^ or semiconducting materials.^66,67^ Second, a significant limitation exists in the functionalization of the DNA tile-covered substrate. To functionalize DNA materials, it is essential to form structures that contain attachment sites. By using this design strategy, DNA tile-based structures and DNA origami structures^68^ have enabled the placement of a wide variety of materials, such as small proteins,^26,45^ DNA,^69,70^ RNA,^71^ AgNP,^72^ AuNP,^73–76^ and chemicals,^77–80^ at specific positions on the fabricated structures. Nevertheless, this approach has not been applied to DNA surface-assisted growth, which cannot form structures in directions vertical to the surface providing the attachment site. As such, research aimed for extended applications is so far limited, with only a few studies reporting the application of DNA surface-assisted growth, such as an insulating layer for solar cells.^81^ Although previous studies have acknowledged these challenges and suggested improved methods, no fully effective solution has emerged. In particular, high coverage rate of large-scale vertical arrays on macroscale surfaces has proven difficult, with prior efforts achieving about 80% efficiency.^82^

In this regard, the growing and homogeneous distribution of vertically oriented attachment sites on DNA tiles equips DNA tile-based constructs for uniform functionalization at the layer level. To facilitate this goal, we have developed advanced DNA tile surface-assisted growth to enable DNA strands to assemble on the surface and form vertical attachment sites on the tile. Our strategy involves two new stepwise surface-assisted growth methods: (1) growing individual tiles on the surface through low-temperature thermal annealing after initial thermal annealing, and (2) covering the surface with a DNA crystal structure formed through initial thermal annealing, followed by low-temperature thermal annealing to induce rearrangement. Using these new surface-assisted growth methods, we designed double-crossover (DX) tiles to control the distance between attachment sites at nearest neighbor distances of 4, 8, and 12 nm, demonstrating the possibility of pattern control in crystals through atomic force microscopy (AFM) imaging. AFM is ideal for analyzing DNA structure since it allows for the characterization of surface structures with controlled composition in aqueous media from the nano- to the microscale.^83,84^ Our observations open the unique possible to create macro-scale large-area arrays of vertical structures with nanoscale controllability, providing highly defined functional attachment sites.

## Results and Discussion

### Design Principle and Structural Characterizations

To fabricate vertical DNA nanostructure arrays on surfaces, we employed DX tiles,^85^ which self-assemble based on the programmable characteristics of DNA.^86,87^ These DX tiles are formed by the self-assembly of 4–6 different strands, each ranging from 26 to 48-nucleotide-long, into a defined tile shape. By repeatedly attaching these identical tiles following predetermined binding rules, approximately 1000 to 2000 tiles join to form a higher-order crystal structure.^88^ We refer to these identical tiles as “unit-tiles”. The unit-tiles can be divided into two categories based on the presence of vertical structures that provide an 8-nucleotide receptor attachment site: a tile without vertical structures (Figure 1a, depicted as a brown hexagonal prism) and a tile with vertical structures to tile (Figure 1a, an ivory hexagonal prism). By defining the unit-tiles using these two types, the design aids in controlling the distance between the receptor attachment sites.

**Figure 1:**
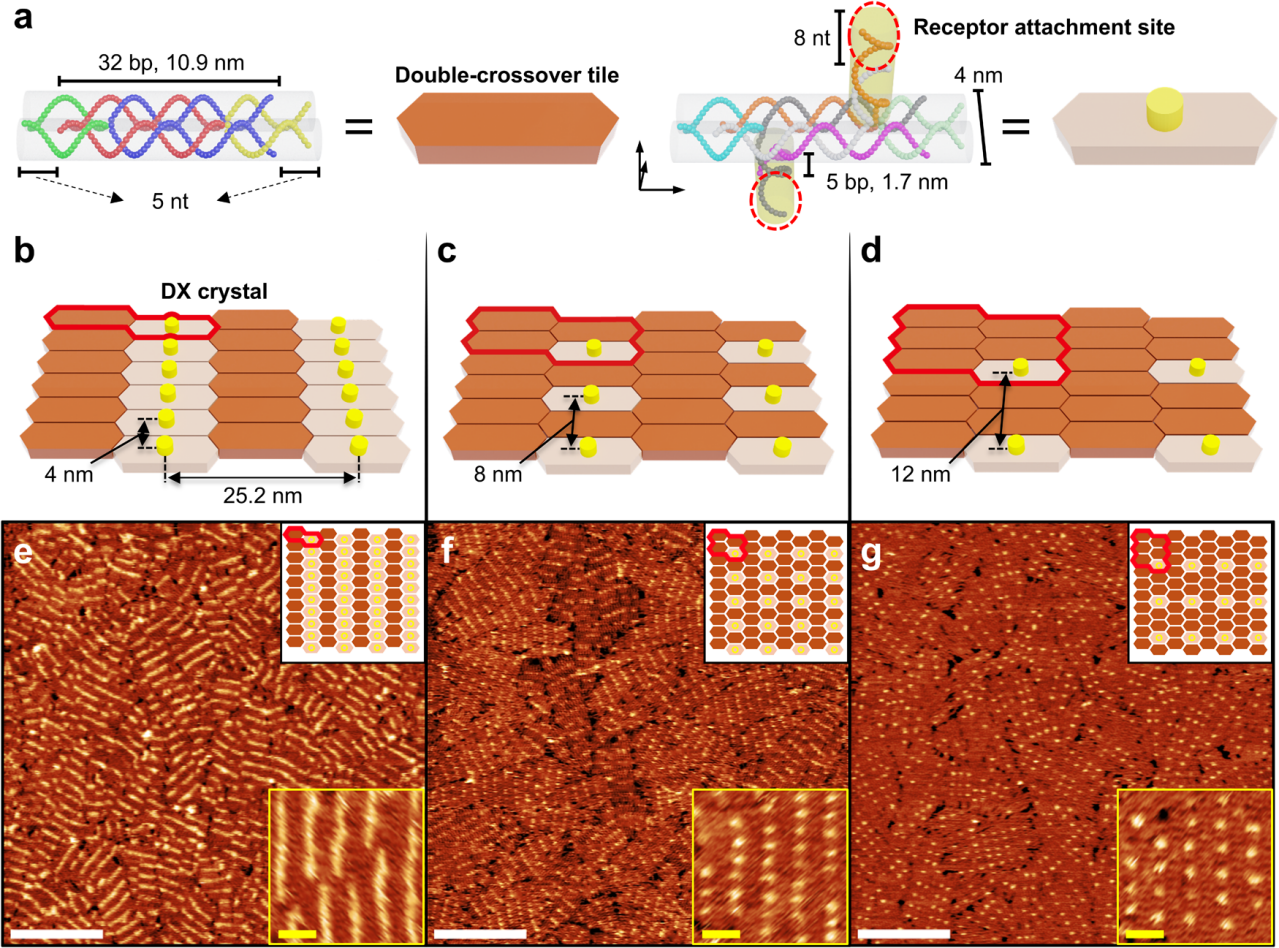
Schematics and AFM images of vertical structure array surfaces. (a) Schematic illustration of the DX tiles used in the experiments. The chains of different colors represent distinct nucleic acid strands. These strands form DX tiles, represented as hexagonal prisms. A DX tile without a vertical structure on the tile is depicted as a brown hexagonal prism, while a DX tile containing a vertical structure on the tile with an attachment site (8-nucleotide, shown in a red circle) is illustrated as an ivory hexagonal prism with a yellow cylinder (right). (b–d) The DX tile forms a higher-order crystal structure by the repetitive combination of unit-tiles according to the design. Red outlines show the unit-tiles of each higher-order crystal structure: (b) Two unit-tiles (2-tile): One brown and one ivory hexagonal prism; (c) Four unit-tiles (4-tile): Three brown and one ivory hexagonal prisms; (d) Six unit-tiles (6-tile): Five brown and one ivory hexagonal prisms. (e–g) AFM images of the crystal structure correspond to the schematic illustrations above. The black inset boxes illustrate the DX crystal structure according to the unit-tiles, while the yellow inset box displays a magnified AFM image to observe the DX crystal structures at the tile level (white scale bars: 200 nm; yellow scale bars: 30 nm).

One DX tile theoretically has a length of 12.6 nm along the helical direction and a geometric size of 4 nm in the longitudinal direction, which is perpendicular to the helical axis, consisting of the thickness of two duplexes.^89^ Consequently, unit-tiles are arranged diagonally, and the nearest-neighbor receptor attachment sites of the unit-tiles are as follows, depending on the design. Two unit-tiles (Figure 1b, hereafter referred to as 2-tile) consist of one tile and a vertical tile, with a spacing of 4 nm. Four unit-tiles (Figure 1c, referred to as 4-tile) include three tiles and a vertical tile, with a spacing of 8 nm. Six unit-tiles (Figure 1d, referred to as 6-tile) are made up of five tiles and a vertical tile, with a spacing of 12 nm.

The structure providing the receptor attachment site, positioned in a direction vertical to the tile, is composed of a 5-base pair section that offers robust structural stability for each array,^90^ topped with 8 unpaired nucleotides. DX crystals containing these designed structures were fabricated in solution without any support substrate through thermal annealing and their structures were confirmed using AFM. In the AFM images, higher structures appear brighter, and by measuring the spacing between these bright tiles and dark tiles, it was confirmed that the tiles and vertical structures are formed according to defined regulations (Figures S2–S4).

Although we evidenced that the designed structures without a supporting substrate were successfully fabricated, applying the mica-assisted surface growth method to cover the surface on a macro-scale did not result in growth following the predefined tile binding regulations. Consequently, the structures were fabricated using the stepwise thermal annealing method described later, successfully covering a 25 mm^2^ surface (Figure 1e–g).

The mica surface mostly covered with DX crystal structures formed by surface-assisted growth, was verified through AFM imaging. On the surface of the 2-tile, due to the resolution limits of the AFM probe and the convolution effect, the array of vertical structures providing the receptor attachment site appeared as bright stripes in the AFM image (Figure 1e). The AFM image of the 4-tile design, based on a wider spacing of vertical structures than the 2-tile, showed individual bright spots instead of bright stripes (Figure 1f). Additionally, the 6-tile design, with even larger spacing than the 4-tile, displayed more widely spaced bright spots (Figure 1g).

The DX crystal structures fabricated on the surface using the stepwise surface-assisted growth method contained domains that were, on average, 44% smaller than the DX crystal structures fabricated without a supporting substrate (Figure S5). AFM imaging confirmed a uniformly covered surface on a millimeter-scale mica substrate, with consistent results observed at various locations on the substrate (Figures S6–S8). This uniformity displayed an average coverage of 98.2 ± 1.3%, which is in line with the around 98.6 ± 0.9% coverage seen in previous studies optimized for substrate-assisted growth, and similar to other surface-assisted grown DX crystal structures.^91^

### Advanced Surface-Assisted Growth Method

The thermal annealing process for DX crystal structures progresses by cooling a solution containing the DNA components to room temperature at a temperature higher than the melting temperature of the designed nanostructures. During this cooling process, the DNA components self-assemble. More specifically, distinct nucleic acid strands form unit-tiles according to Watson–Crick base pairing, and individual tiles subsequently join into higher-order crystal structures according to predetermined regulations. The surface-assisted growth method involves the introduction of the supporting substrate during the thermal annealing process. This method utilizes electrostatic interactions among the negatively charged supporting surface, the negatively charged DNA backbones, and the positive ions in the DNA nanostructure buffer solution to conduct the thermal annealing process on a two-dimensional (2D) supporting substrate, rather than in a three-dimensional solution space. DX crystal structures formed via surface-assisted growth appear to follow a similar self-assembly process without the need for a supporting substrate, where strands form tiles, tiles form crystal structures, and the rearrangement of tiles at the edges of the crystal domains ultimately covers the surface. This method allows for the formation of DX crystal structures with low nucleation density and high surface coverage on macro-scale surfaces.^42,92^

The fabrication of uniform nanostructures on a macro-scale surface in a designed pattern has been of keen interest, combining the nanoscale controllability of DNA with its macro-scale uniformity.^93^ However, it has been challenging to form structures in the direction vertical to the surface, making it difficult to utilize the advantages of large-area surface functionalization or enabling nanoarrays fabrication.

Previous literature indicated that tiles with vertical structures are difficult to form through surface growth and can only be fabricated under limited conditions, due to the interference caused by geometrical hindrance between the backbone and the surface, which weakens the interaction between the surfaces.^91^ We hypothesized that, in addition to the hindrance between the vertical tiles and the surface, tile assembly on the two-dimensional substrate might also be an unknown obstacle in the formation process of a tile with a vertical structure. To address this challenge, we developed and validated the surface-assisted growth method using three different approaches: (1). The first method involved thermal annealing with distinct strands that constitute the structures and the supporting substrate, similar to the previously reported method (Figure 2a, Method I). (2). The second thermal annealing approach aimed to avoid undesirable hindrances during the tile formation process on the surface, by preparing individual tile structures beforehand and subsequently annealing these prepared tiles with the supporting surface at temperatures from 40 °C to room temperature to prevent tile deformation (Figure 2b, Method II). (3). The third approach conducted both the tile formation and the crystal formation process without a supporting substrate, thereafter transferring the completed crystal structure onto the surface, performing rearrangement, and finally annealing at temperatures from 40 °C to room temperature to prevent tile deformation (Figure 2c, Method III).

**Figure 2:**
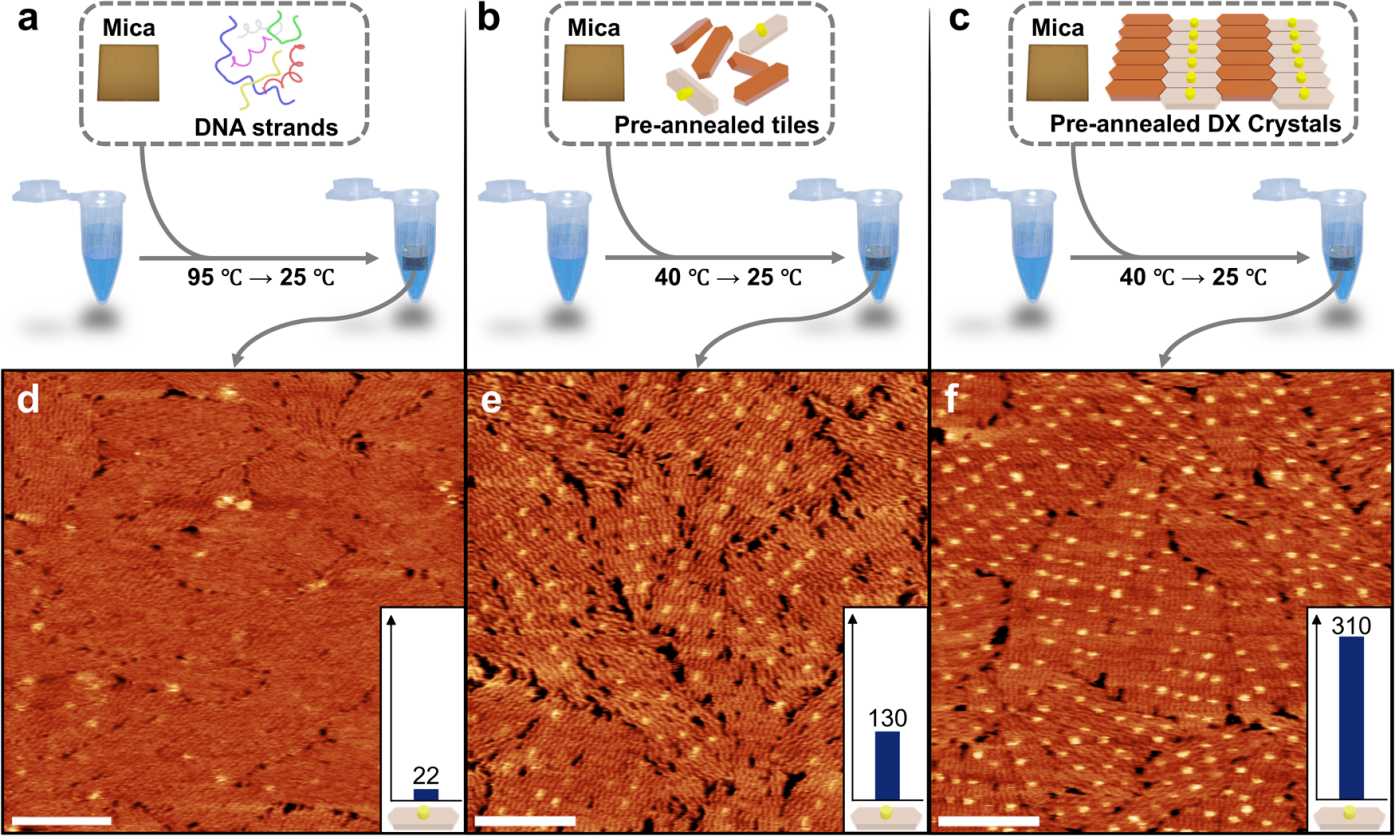
Three different surface-assisted growth methods for vertical structure array surfaces. (a) Method I: Thermal annealing from 95 to 25 °C with DNA strands (colored chains) forming the DX crystal structure and the substrate (ochre-colored square). (b) Method II: Thermal annealing from 40 to 25 °C with separately prepared individual tiles (brown and ivory hexagonal prisms) assembling the DX crystal structure and the substrate. (c) Method III: Thermal annealing from 40 to 25 °C with prepared crystal structures (crystal consists of brown and ivory hexagonal prisms) and the substrate. (d–f) AFM images of the crystal structure correspond to the methods above. Inset bar graphs show the number of receptor attachment sites for each AFM image (scale bar: 100 nm).

We specifically used the 6-tile sample to test the three methods (Method I, II, III), because it showed the highest number of unit-tiles and relatively few tiles with vertical structures (one of six unit-tiles), making it straightforward to verify compliance with the unit-tile binding regulation. The AFM image in Figure 2d reveals that the surface fabricated using Method I rarely self-assembles tiles containing vertical structures (visible as bright dots). Precisely, only 22 tiles were formed, which corresponds to about 4.1% of the theoretical maximum number of tiles containing vertical structures within the scan area (0.25 μm^2^), given the unit-tile size of approximately 467 nm^2^. Since these structures were primarily found at the edges of crystal domains, we assume that the remaining tiles containing vertical structures in solution adhered to the surface during the rearrangement process after crystallization.

Method II, in which we introduced stepwise thermal annealing with a supporting substrate based on prepared individual tiles, displays significantly more bright spots when compared to the results of Method I. In detail, the AFM data in Figure 2e show that 130 vertical structures were formed, representing about 24.3% of the theoretical maximum.

Finally, in Method III thermal annealing is conducted by placing the fabricated crystal structure on the substrate and lowering the temperature from 40 to 25 °C, a range below the melting temperature of the tiles, but still sufficient to deform and reassemble tile connections (Figures S9–S11).^94^ This method was designed to compensate for the low mobility observed in Method II, which is affected by the charge interactions between the supporting substrate and the tiles. By using crystal structures prepared in solution and preformed small tile nucleation seeds, we anticipated that growth and rearrangement would result in a substrate following tile binding regulations with higher accuracy. Indeed, the AFM image in Figure 2f confirmed that Method III formed the highest number of tiles containing vertical structures among the three methods. A total of 310 vertical structures were formed, representing 58.1% of the theoretical maximum. (Additional AFM data of the DX surface fabricated using Method I and II are shown in the Supporting Information (Figures S12–S17). Furthermore, changes in domain size due to repetitive thermal cycling and varying Mg^2+^ concentrations are shown in the Supporting Information (Figure S18–S19)).

### Functionalization of DX Surfaces

To demonstrate the functionalization capabilities of the fabricated nanostructure arrays, we ligated a thrombin binding aptamer,^95^ incorporating structures for signal reporting (TBA15), to the vertical arrays on the surface for providing spatially defined thrombin binding sites. Figure 3a depicts the stepwise thermal annealing process of the DX surface, involving the hybridization of TBA15s onto the attachment sites. After completing the thermal annealing of the DNA tile surface, the TBA15 strands with 8 unpaired nucleotides for hybridization with an attachment site were added, heated up, and subsequently cooled down from 28 to 25 °C, so as to facilitate the hybridization of TBA15s.

**Figure 3:**
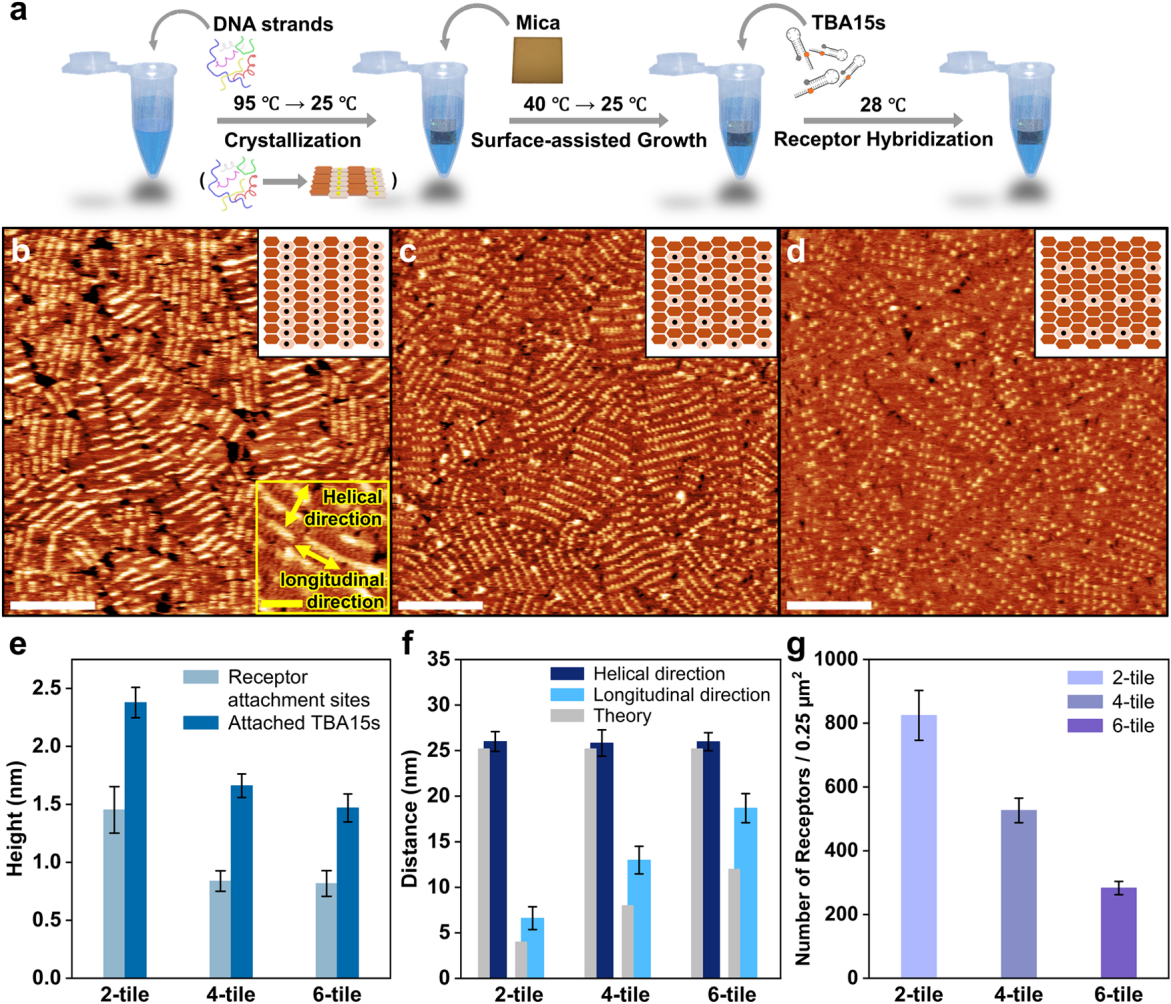
Characterization of TBA15 functionalized vertical structure arrays on the surfaces. (a) Schematic image of the thermal annealing process of the TBA15 array. AFM images of the TBA15 arrays on the (b) 2-tile, (c) 4-tile, and (d) 6-tile surfaces, respectively. The black inset boxes illustrate the DX surfaces and the dots on an ivory hexagonal prism represent the attached TBA15 on the DX tile. (e) Bar graphs of the average heights of vertical structures, each with an attachment site and TBA15s attached vertical structures. (f) The average of 50 measured distances between nearest-neighbor TBA15s in each direction, like those indicated in the yellow inset box in (b). (g) The average number of TBA15s in 0.25 μm2 areas. (white scale bars: 200 nm; yellow scale bars: 50 nm).

Figure 3b–d represent the AFM data for TBA15-functionalized 2-tile, 4-tile, and 6-tile surfaces. The brighter and thicker stripes and spots, when compared to the unfunctionalized DX surfaces (Figure 1e–g), can be clearly discerned. After functionalizing the surface with TBA15s, the height differences were precisely determined through height analysis of the AFM images. The bar graphs Figure 3e graphs display the height differences between the vertical structures containing attachment sites and the TBA15-functionalized vertical structures on the surfaces (in over 50 AFM height profiles each). The bar graphs indicate that the height of the TBA15-functionalized structures is on average 1.8 times larger than that of the bare attachment sites.

The average height of the structure functionalized with TBA15 was measured to be 2.4 nm on the 2-tile. Due to the soft nature of the material in a liquid environment during intermittent contact AFM measurements, this experimentally determined value was lower than the theoretical of 9.8 nm for a vertical structure containing TBA15.^96,97^ Additionally, we observed that the heights consistently increased in the order of 6-tile, 4-tile, and 2-tile. According to the entropy brush effect,^98,99^ vertical structures exhibit a behavior that increases entropy and maximizes spatial occupancy. Therefore, in the lower density 4- and 6-tile systems, there is relatively higher spatial occupancy compared to the 2-tile system. This leads to an increase in entropy and a higher degree of geometrical freedom, resulting in lower rigidity and lower heights in AFM analysis. In contrast, the 2-tile, which shows higher density, shows tight interactions between vertical structures, causing them to compress. This leads to relatively lower entropy and a reduced degree of geometrical freedom, resulting in increased rigidity and higher heights in AFM analysis. For all tile structures, the surface roughness increased with the attachment of TBA15 (Figure S20).

In the AFM image of the TBA15-functionalized 2-tile surface (Figure 3b), the bright stripes represent pillars of TBA15s. These pillars were aligned along the helical direction of the duplexes, separated by 25.8–26 nm, which is remarkably close to the designed distance of 25.2 nm. Along the longitudinal direction perpendicular to the helical direction, the average measured distance of 6.6 ± 1.2 nm was longer than the expected spacing of 4 nm (width of two duplexes). This difference arises from the electrostatic repulsion between the DNA phosphate backbones, which repels the DNA duplexes from each other, resulting in increased distances in the longitudinal direction.^100^ The same phenomenon has been observed in previously reported DNA nanostructures.^68,87^ Likewise, the 4-tile and 6-tile surfaces (Figure 3c,3d) also display increased distances when compared to the theoretical design (Figure 3f).

From Figure 3g, the average number of TBA15 molecules in 0.25 μm^2^ areas for each type of unit-tile design was calculated out of more than 12 images each. Thereby a height increase of at least 60% was taken as the criterion for TBA15 binding. The 2-tile revealed 824 ± 78 molecules, the 4-tile 526 ± 39 TBA15s, and the 6-tile 283 ± 21 TBA15s, all indicating a satisfactory high density. Although there were some variations with respect to the three different numbers of tiles per unit, they were within an acceptable standard deviation (e.g., 64% of 4-tile when compared to the 2-tile design, with a standard deviation of 7.3%). Conclusively, from these number density calculations, almost all attachment sites appeared to have hybridized TBA15s and no pristine attachment sites were observed in the over 50 analyses of the images for the 2-, 4-, and 6-tile designs. (Figure 3b–e). (Additional AFM data of TBA15-functionalized surfaces can be found in Supporting Information (Figures S21–S23)). Although 2, 4, and 6-tile appear to fully cover the surface in AFM images, there is an average discrepancy of 37.4% between the expected and observed numbers of vertical structures (calculated by dividing the total surface area (2500 nm^2^) by the area of a unit-tile (experimental yield): 58.6% for 2-tile, 70.3% for 4-tile, 58.8% for 6-tile). The theoretical maximum numbers are calculated under the assumption that the domain is a single, unified area, with a base distance of 0.34 nm, and a duplex width of 4 nm. The observed difference arises from electrostatic repulsion between tile structures and the absence of structures near boundaries resulting from differences in domains. This level of yield was achieved when examined locally within an area of 2500 nm^2^, however, when observed in larger scale, vacant spaces at the interfaces where different domains reach may lead to a decrease in the overall yield.

### Fluorescence Signal Characterization

TBA15 is a 15-nucleotide-long DNA aptamer with a binding affinity of *K*_D_ ∼ 70–100 nM for thrombin exosite I.^95,101,102^ To exploit binding the high binding affinity of TBA15, we performed a characterization of TBA15-functionalized surfaces by introducing thrombin molecules onto the surfaces to induce the binding-induced conformational change in TBA15. To achieve this goal, we designed TBA15 to attach to vertical structure arrays, allowing it to bind thrombin and emit a fluorescence signal. Upon thrombin binding to TBA15, TBA15 transforms into a G-quadruplex. This secondary structure is formed in nucleic acids rich in guanine and consists of stacked sets of four guanine bases,,^103–105^ which releases out the quencher-modified detachment strand, thereby resulting in fluorescence emission (Figure 4a). It has been reported in previous studies that aptamers with high binding affinity can disrupt DNA hybridizations and release an incumbent strand upon molecular binding.^106,107^

**Figure 4:**
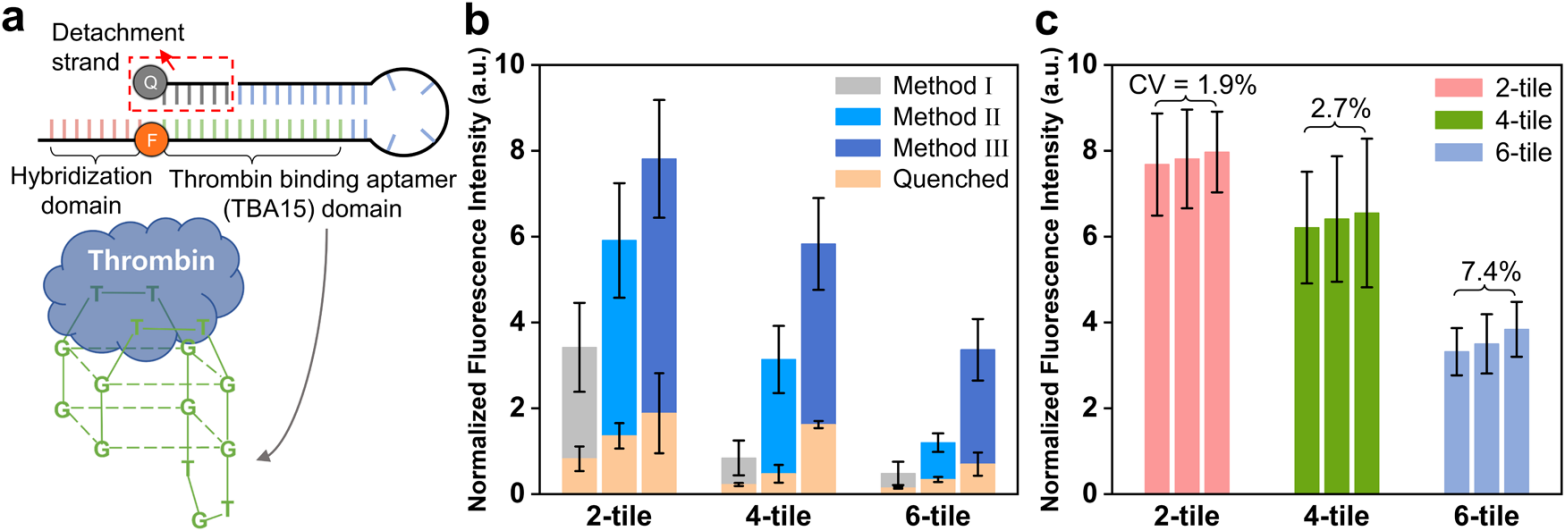
Characterization of the fluorescent signal from TBA15-functionalized surfaces. (a) Schematic depiction of TBA15, including unpaired nucleotides for hybridization with an attachment site and modifications for selective fluorescence emission. The red-colored sequences represent the hybridization domain (8 nt) complementary to the sequences of an attachment site of vertical structure arrays. The green-colored sequences represent the TBA15 sequences (15 nt), with a fluorophore (TAMRA) modification. The gray-colored sequences represent the detachment strand (6 nt), with a quencher modification at the 3′-end. Once thrombin binds to TBA15, it releases out the detachment strand with the quencher (red dashed box). (b) Fluorescence intensity of TBA15-functionalized surfaces for each unit-tile and method, with thrombin at a concentration of 200 nM. Each orange-colored bar graph indicates the quenched fluorescence intensity under the given conditions. (c) Each bar graph corresponds to the fluorescence intensity of a single functionalized surface following a unit-tile design. The coefficient of variation (CV) is shown for each unit-tile surface.

Depending on the concentration of Mg^2+^ in the buffer solution, which stabilizes the DNA nanostructure,^92,108–110^ the binding of TBA15 and thrombin gradually displaces the quencher-modified detachment strands. Consequently, the fluorescence intensity of TBA15 increases over 12 h and quenched TBA15 shows significantly low fluorescence intensity compared to intensity shown when thrombin introduced (Figure S24). To ensure completion of the reaction, TBA15-functionalized surfaces and thrombin were incubated for 24 h, after which the fluorescence intensity was finally measured (Figure 4b).

The bar graphs in Figure 4b present the fluorescence intensities of the three different unit-tile surfaces for the three proposed thermal annealing methods. Assuming that all TBA15 structures emit fluorescence upon introduction of thrombin under the given conditions, the normalized fluorescence intensity proportionally reflects the amount of TBA15 attached to the surface. The data reveal that Method III exhibited the highest intensity over all three different unit-tile surfaces. Regarding the functionalization yield, this finding highlights that Method III is much more effective with its complex designs featuring a higher number of unit-tiles by minimizing geometrical hindrance during both tile formation and crystal growth. In stark contrast, for Method I, which was primarily used in previous surface-assisted growth methods, the intensity is markedly smaller, even with the complexity of a 4-tile design.

To demonstrate surface-to-surface reproducibility, the fluorescence intensity of three identical DX surfaces, fabricated using Method III for each unit-tile surface, were compared. In Figure 4c, the bar graphs display the normalized fluorescence intensity for each TBA15-functionalized surface depending on the unit-tile design. The results show low coefficient-of-variation (CV) values of 1.9, 2.7, and 7.4% for the 2-tile, 4-tile, and 6-tile designs, respectively. Given that the typical CV for signal reproducibility of fluorescence biosensors ranges from 5–20%,^111–113^ our results reveals that the DX surfaces produced using Method III has achieved a comparable CV level, even at a macro-scale production level.

## Conclusions

In this study, we have demonstrated the feasibility of using DNA tile-based structures to achieve macroscopic large-area crystal growth with vertically oriented structure arrays, which provide attachment sites for functionalization and precise control over the spacing of these sites. Our results indicate that functionalization associated with DNA surface-assisted growth can be effectively addressed through new stepwise thermal annealing methods. These methods enable the macroscopic fabrication of vertical structure arrays with nanoscale controllability. Our advancement opens up potential applications for functionalizing DNA nanostructures in areas requiring extensive surface coverage. Surface immobilization and arraying of bioprobes can achieve higher sensitivity at lower costs. Previous studies have demonstrated that aligning the orientation of receptors^114^ and preventing inactive receptors caused by random orientation effectively enhances sensitivity.^115^ Here, we developed a receptor surface with high density to be utilized as a multiaptamer platform by adjusting the spacing between aptamers. One key feature of the increased sensor capacity offered by an aptamer array is the capability of the sensor to capture many target molecules simultaneously. This larger capacity translates into an overall higher signal, improving the signal-to-noise ratio.^116,117^ In addition, because binding events are distributed across numerous sites with defined distances, the signal is less prone to stochastic fluctuations that can dominate in single-site systems.

Such arrays can also serve as sensing platforms in Surface Plasmon Resonance (SPR) that is widely used for real-time,^118^ label-free detection of molecular interactions^119^ and in various AFM-based applications, and are compatible with single-molecule force spectroscopy^83^ and fluorescence microscopy detection,^120^ where sensitivity can be enhanced or distance modulation is required for analysis.^121^ Our work demonstrates that DNA nanotechnology, with its inherent nanoscale controllability, can be effectively scaled up to create functional surfaces that are both uniform and versatile.

## Materials and Methods

### Materials

All synthetic oligonucleotides including fluorophore (TAMRA)-modified, and quencher (BHQ2)-modified strands were purchased from Bioneer (Daejeon, Korea) and purified by standard purification (bioRP), and polyacrylamide gel electrophoresis (PAGE) for modified strands. The details of the strand sequences can be found in the Supporting Information (Figure S1). Thrombin from human plasma (T6884) was purchased from Sigma-Aldrich.

### Design of DX Tiles

SEQMAK software^122^ was used for designing base sequences of all DX tiles. All individual tiles were designed to avoid repetition within a 6-nucleotide-long sequence length, and the entire strand forming the DX crystal was configured to avoid repetition within an 8-nucleotide-long sequence length.

### Formation of DX Surface

The strands of each DX surface were mixed stoichiometrically in an equimolar ratio in a 1.5 mL Eppendorf tube with tris-acetate-EDTA/Mg^2+^ buffer (1× TAE/Mg^2+^; 40 mM Tris, 1 mM EDTA and magnesium tetrahydrate 12.5 mM, pH 8.0). Mixed strands were gradually annealed from 95 to 25 °C for 40 h in a 1.5-L water bath insulated in a styrofoam box. After 95 °C annealing, freshly cleaved mica substrates are placed inside the mixed solution for surface-assisted growth, followed by the second thermal annealing at 40 to 25 °C for 24 h. The initial temperature for the second thermal annealing was optimized between 55 and 40 °C. The concentrations of 2, 4, and 6-tile DX surface solutions were 50, 35, and 25 nM and the volume of all solutions was 200 μL.

### Attachment of TBA15

Fluorophore-modified strands containing the TBA15 base sequence and quencher-modified detachment strands were mixed in equimolar ratios in an Eppendorf tube and annealed from 95 to 25 °C with 1× TAE/Mg^2+^ buffer. Then, the mica substrate covered with the DX surface was placed in the TBA15 solution for hybridization of the receptor attachment site on the DX surface and the hybridization domain in TBA15 at 28 °C for 24 h. The concentration of the TBA15 solution was 200 nM, and the volume of the solution was 200 μL. After hybridization, all samples were washed and stored in a 1× TAE/Mg^2+^ buffer.

### AFM Imaging

For AFM measurement, DNA-functionalized mica substrate was attached to the sample stage using epoxy adhesive (Araldite, Huntsman Advanced Materials, Basel, Switzerland), and 900 μL 1× TAE/Mg^2+^ buffer was filled in the fluid cell of the sample stage. All AFM images were obtained using AC mode (9500, Keysight Technologies, Santa Clara, CA) with PEAKFORCE-HIRS–F-B cantilever (0.1 N/m nominal spring constant, Bruker, Camarillo, CA). The resonance frequency of the cantilever was selected between 15–30 kHz, with free amplitude usually less than 5 nm to minimize force on the surface structure. Imaging was performed at scan rates between 0.8–2.0 Hz with 512 pixels per line. All AFM images were analyzed using open source software Gwyddion.^123^ ImageJ software was used to operate the surface coverage analysis of AFM images.

### Fluorescence Measurement

Samples were prepared on a glass slide with 100 μL of 1× TAE/Mg^2+^ buffer. The laser spot (CWDPSS 532 nm laser, Pavilion Integration Corp., San Jose, CA) was located and focused on the sample through a 10× objective lens of an optical microscope. Under the focused laser beam of 17.4 μW, the fluorescence emission spectrum was measured by an EMCCD (DU970P, Andor, Belfast, Northern Ireland) equipped with a spectrograph (Shamrock 303i, Andor). The emission was measured with an exposure time of 10 s for all samples.

## Supplementary Material



## Notes

DNA sequence details and additional AFM images (PDF)
