# Supplementary material for: Vertical DNA Nanostructure Arrays: Facilitating Functionalization on Macro-Scale Surfaces
Source: ACS Nano. 2025 Apr 9;19(20):19353–63. doi: 10.1021/acsnano.5c03100 (PMC12120987; doi:10.1021/acsnano.5c03100)
Supplement: Supplementary file 1 [file nn5c03100_si_001.pdf]

Supporting information for

Vertical DNA Nanostructure Arrays: Facilitating Functionalization on Macro-Scale Surfaces

Hyeonjun Kwon[1†], Jihoon Shin[1†], Siqi Sun[1], Rong Zhu[2], Sarah Stainer[2], Peter Hinterdorfer[2], Sang-Joon Cho[3], Dong-Hwan Kim[1], Yoo Jin Oh[2*]*

[1] *School of Chemical Engineering, Sungkyunkwan University, Suwon 16419, Republic of Korea*

[2] *Department of Applied Experimental Biophysics, Institute of Biophysics, Johannes Kepler University Linz, Gruberstrasse 40, A-4020 Linz, Austria*

[3] *Park Systems, Corp., KANC 15F, Gwanggyo-ro 109, Suwon 16229, Republic of Korea*

Chapter 1. DNA strand sequence.

- Sequence map of the designed double-crossover tiles (Figure S1).
- Sequence details for oligonucleotide pool and functional strands (Table S1).
- Unit-tiles composition of the crystal structure (Table S2).

Chapter 2. Additional AFM analysis of DX crystals and surfaces

- Characterization of DX crystal structures fabricated in solution (Figure S2–4).
- Domain size of DX crystals depending on the supporting substrate (Figure S5).
- AFM images of DX surfaces in various locations on the substrate (Figure S6–8).
- Analysis of the influence of temperature on surface-assisted growth (Figure S9–11).
- AFM images of DX surfaces fabricated using Methods I and II (Figure S12–17).
- Analysis of the change in domain size in different condition (Figure S18–19).
- Changes in the roughness of three DX surfaces due to TBA15 attachment (Figure S20).
- AFM images of TBA15-functionalized surface in various locations (Figure S21–23).

Chapter 3. Fluorescence Characterization

- Fluorescence signal intensity of TBA15 after thrombin introduction (Figure S24).

Chapter 1. DNA strands sequences

The sequences of all the complexes used in the experiments are provided below. First, a sequence map is shown, followed by a table listing the individual strand names, sequences, and lengths.

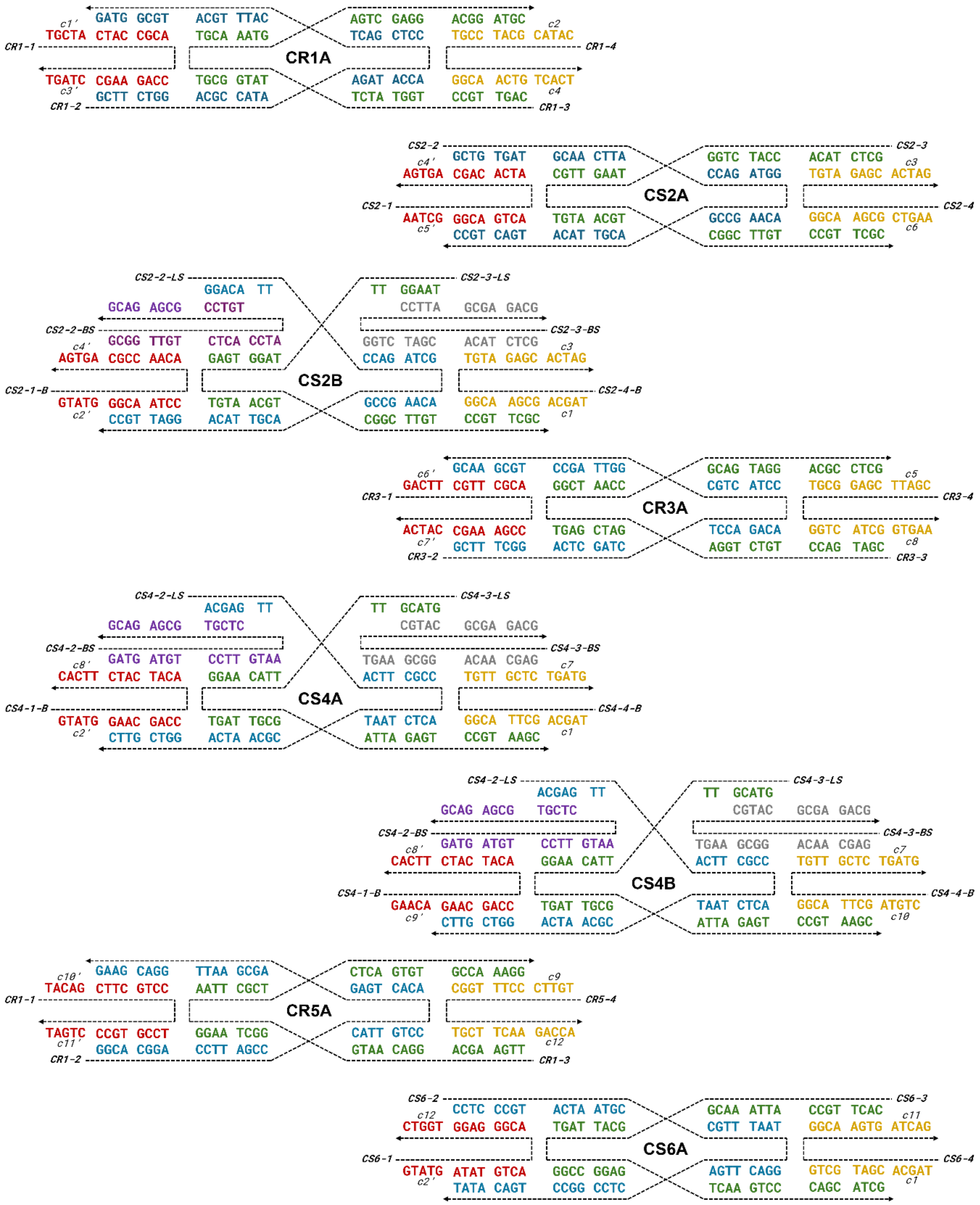

Figure S1. Sequence map of the designed double-crossover tiles. The 5-nucleotide sticky ends of each tile are labeled as c1–c12 and c1′–c12′. All labels are designed to be complementary to the same number labels with an apostrophe, allowing for binding regulation. Sequence details are shown in Table 1.

Tile	Strand	Length (nucleotides)	Base sequence (5′ - 3′)
CR1A	CR1-1	26 nt	TGCTA CTAC CGCA CCAG AAGC CTAGT
	CR1-2	48 nt	GCTT CTGG ACGC CATA AGAT ACCA CCTC GACT CATT TGCA TGCG GTAG
	CR1-3	48 nt	CAGT TGCC TGGT ATCT TATG GCGT TGCA AATG AGTC GAGG ACGG ATGC
	CR1-4	26 nt	CATAC GCAT CCGT GGCA ACTG TCACT
CS2A	CS2-1	26 nt	AATCG GGCA GTCA ATCA CAGC AGTGA
	CS2-2	48 nt	GCTG TGAT GCAA CTTA CCAG ATGG ACAA GCCG ACGT TACA TGAC TGCC
	CS2-3	48 nt	GCTC TACA CCAT CTGG TAAG TTGC TGTA ACGT CGGC TTGT CCGT TCGC
	CS2-4	26 nt	AAGTC GCGA ACGG TGTA GAGC ACTAG
CS2B	CS2-1-B	26 nt	GTATG GGCA ATCC ACAA CCGC AGTGA
	CS2-2-LS	39 nt	GGACA TT CCAG ATCG ACAA GCCG ACGT TACA GGAT TGCC
	CS2-2-BS	26 nt	GCGG TTGT CTCA CCTA TGTCC GCGA GACG
	CS2-3-LS	39 nt	TAAGG TT TAGG TGAG TGTA ACGT CGGC TTGT CCGT TCGC
	CS2-3-BS	26 nt	GCTC TACA CGAT CTGG CCTTA GCGA GACG
	CS2-4-B	26 nt	TAGCA GCGA ACGG TGTA GAGC ACTAG
CR3A	CR3-1	26 nt	GACTT CGTT CGCA CCGA AAGC CATCA
	CR3-2	48 nt	GCTT TCGG ACTC GATC TCCA GACA CCTA CTGC GGTT AGCC TGCG AACG
	CR3-3	48 nt	CGAT GACC TGTC TGGA GATC GAGT GGCT AACC GCAG TAGG ACGC CTCG
	CR3-4	26 nt	CGATT CGAG GCGT GGTC ATCG GTGAA
CS4A	CS4-1	26 nt	GTATG GAAC GACC ACAT CATC TTCAC
	CS4-2-LS	39 nt	ACGAG TT ACTT CGCC ACTC TAAT CGCA ATCA GGTC GTTC
	CS4-2-BS	26 nt	GATG ATGT CCTT GTAA CTCGT GCGA GACG
	CS4-3-LS	39 nt	GTACG TT TTAC AAGG TGAT TGCG ATTA GAGT CCGT AAGC
	CS4-3-BS	26 nt	GAGC AACA GGCG AAGT CGTAC GCGA GACG
	CS4-4	26 nt	TAGCA GCTT ACGG TGTT GCTC TGATG
CS4B	CS4-1-B	26 nt	GAACA GAAC GACC ACAT CATC TTCAC
	CS4-4-B	26 nt	CTGTA GCTT ACGG TGTT GCTC TGATG
CR5A	CR5-1	26 nt	TACAG CTTC GTCC TCCG TGCC CTGAT
	CR5-2	48 nt	GGCA CGGA CCTT AGCC CATT GTCC ACAC TGAG AGCG AATT GGAC GAAG
	CR5-3	48 nt	TTGA AGCA GGAC AATG GGCT AAGG AATT CGCT CTCA GTGT GCCA AAGG
	CR5-4	26 nt	TGTTC CCTT TGGC TGCT TCAA GACCA
CS6A	CS6-1	26 nt	GTATG ATAT GTCA ACGG GAGG TGGTC
	CS6-2	48 nt	CCTC CCGT ACTA ATGC CGTT TAAT GGAC TTGA CTCC GGCC TGAC ATAT
	CS6-3	48 nt	CACT TGCC ATTA AACG GCAT TAGT GGCC GGAG TCAA GTCC CAGC ATCG
	CS6-4	26 nt	TAGCA CGAT GCTG GGCA AGTG ATCAG
RS3	RS3-TBA	37 nt	CACAC CAACC TTTGG TTGGT GTGGT TGG [Tamra-dT] C GTCTC GC
	RS3-Q	6 nt	[BHQ2] ACCAAC

Table 1. Sequence details for oligonucleotide pool and functional strands. All the sequences are present from 5′-3′ (left to right). CS4A shares four strands (CS4-2-LS, BS and CS4-3-LS, BS) with CS4B.

<table>
<tr><th>Unit-tiles</th><th colspan="12">Tile</th></tr>
<tr><td>2-tile</td><td colspan="6">CR1A</td><td colspan="6">CS2B</td></tr>
<tr><td>4-tile</td><td colspan="3">CR1A</td><td colspan="3">CS2A</td><td colspan="3">CR3A</td><td colspan="3">CS4A</td></tr>
<tr><td>6-tile</td><td colspan="2">CR1A</td><td colspan="2">CS2A</td><td colspan="2">CR3A</td><td colspan="2">CS4B</td><td colspan="2">CR5A</td><td colspan="2">CS6A</td></tr>
</table>

Table 2. Unit-tiles composition of the crystal structure. The 2-tile DX crystal is designed to consist of 10 strands (two tiles), the 4-tile DX crystal is designed to consist of 18 strands (four tiles), and the 6-tile DX crystal is designed to consist of 26 strands (six tiles).

Chapter 2. Additional AFM analysis of DX crystals and surfaces

AFM images of DX crystal structures fabricated in free solution allow for cross-sectional examination of the distances between regularly formed vertical structure arrays.

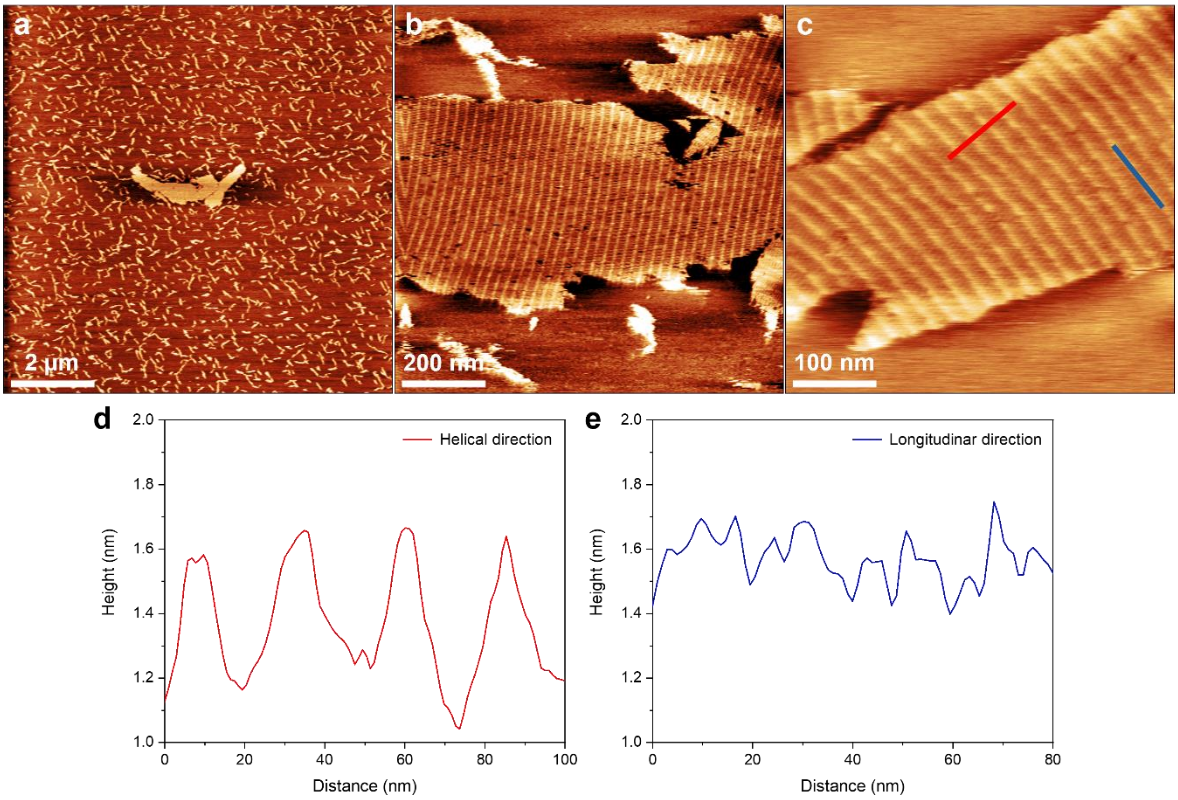

Figure S2. Characterization of 2-tile DX crystal structures fabricated in solution. (a–c) AFM images of 2-tile DX crystals fabricated in free-solution at scan sizes of 10 µm, 1 µm, and 500 nm. Height analysis of 2-tile crystal structure on (d) 100 nm line of helical direction and (e) 80 nm line of longitudinal direction. Vertical structures have an average 25.6 nm distance in the helical direction and an average 6.7 nm distance in the longitudinal direction.

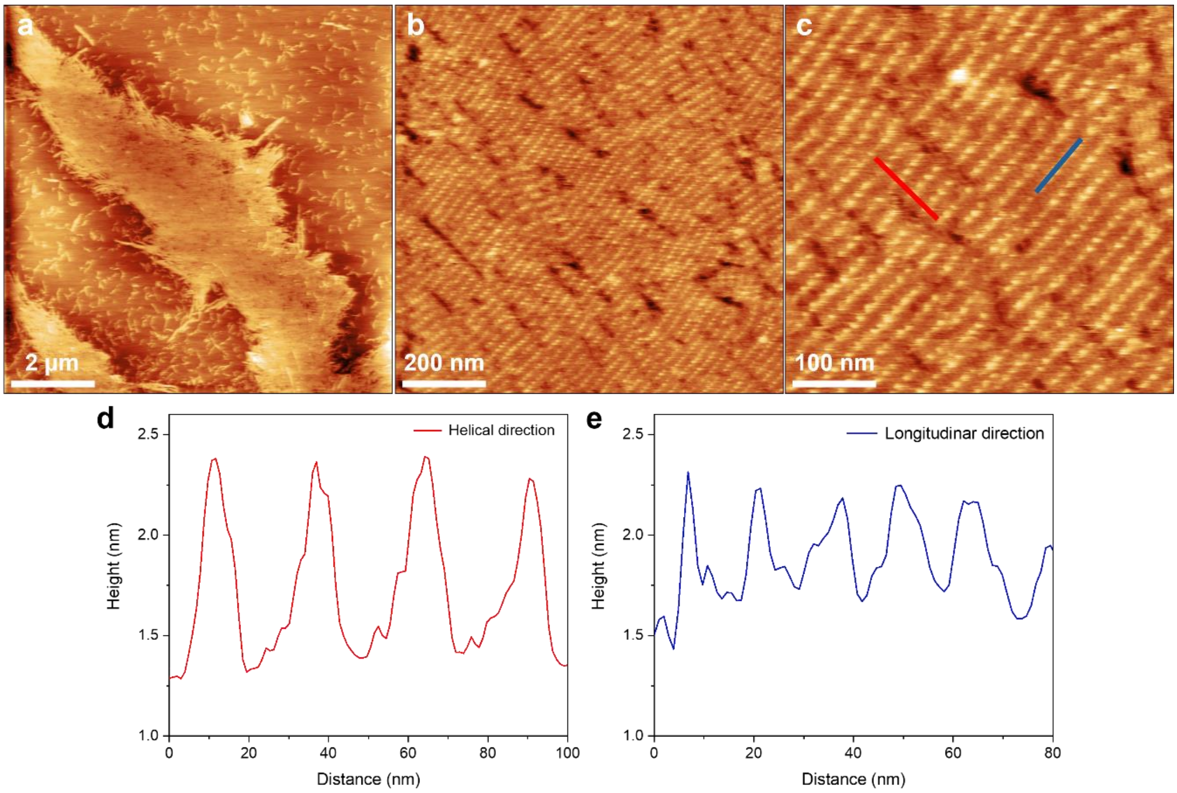

Figure S3. Characterization of 4-tile DX crystal structures fabricated in solution. (a–c) AFM images of 4-tile DX crystals fabricated in free-solution at scan sizes of 10 μm, 1 μm, and 500 nm. Height analysis of 4-tile crystal structure on (d) 100 nm line of helical direction and (e) 80 nm line of longitudinal direction. Vertical structures have an average 25.3 nm distance in the helical direction and an average 13.1 nm distance in the longitudinal direction.

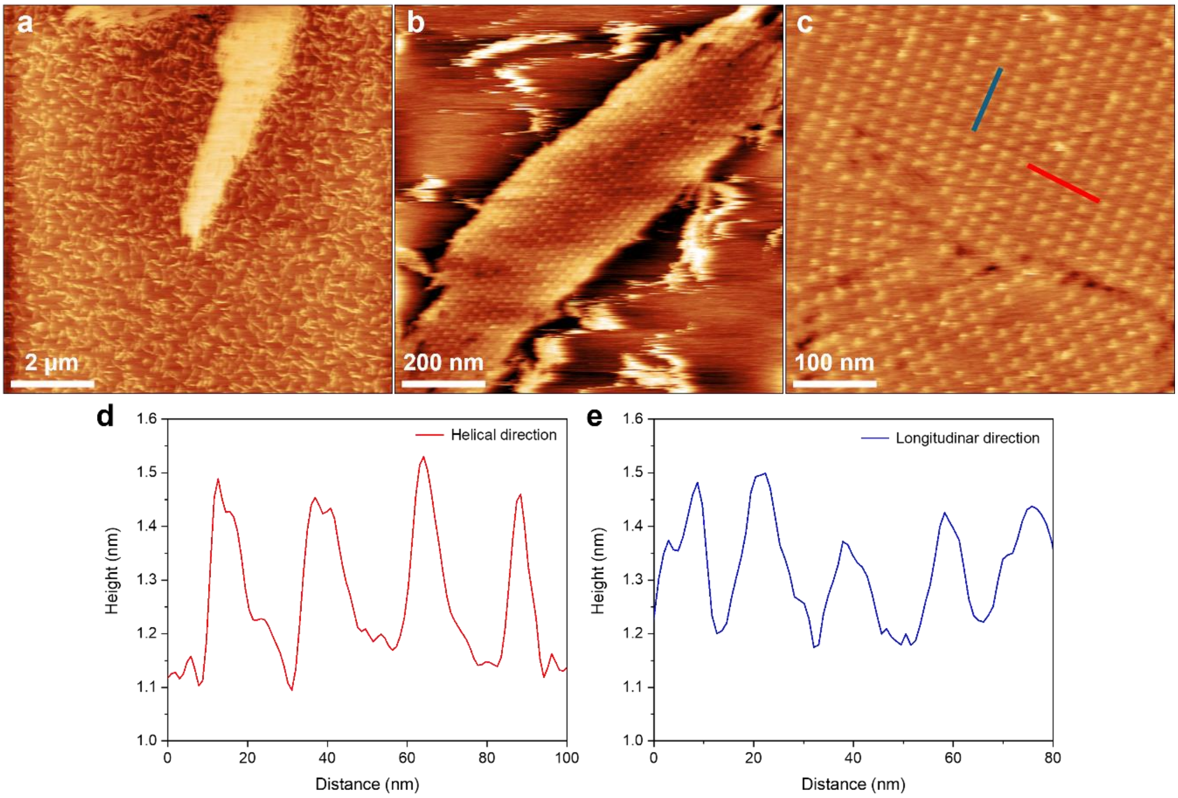

Figure S4. Characterization of 6-tile DX crystal structures fabricated in solution. (a–c) AFM images of 6-tile DX crystals fabricated in free-solution at scan sizes of 10 µm, 1 µm, and 500 nm. Height analysis of 6-tile crystal structure on (d) 100 nm line of helical direction and (e) 80 nm line of longitudinal direction. Vertical structures have an average 25.2 nm distance in the helical direction and an average 17.5 nm distance in the longitudinal direction.

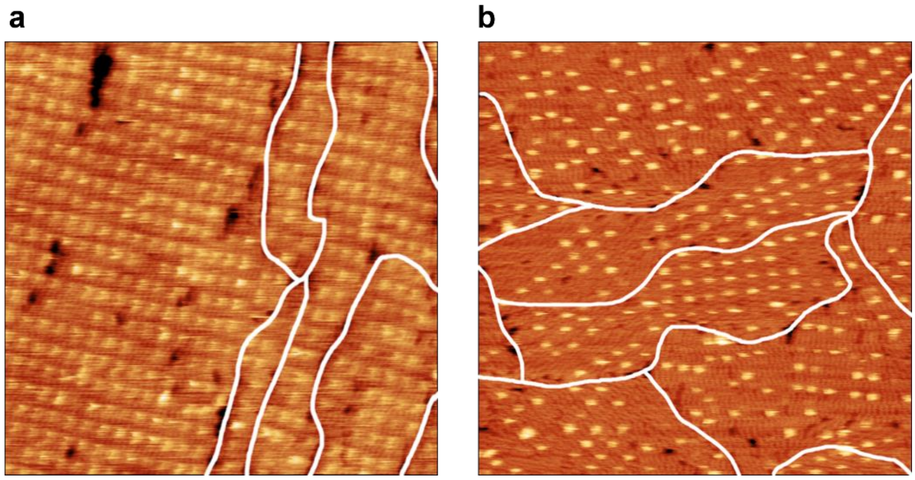

Figure S5. Comparison to domain size of DX crystals fabricated by solution annealing and surface-assisted growth method. (a) Domains of DX crystals fabricated in solution. 0.25 μm^2 area was divided into 7 domains. Domains have an average of 35,700 nm^2 size. (b) Domains of DX crystals fabricated using Method III. 0.25 μm^2 area was divided into 9 domains. Domains have an average of 27,800 nm^2. A similar coverage analysis was conducted in a previous study.[1]

Macro-scale surface formation

AFM images from various locations demonstrate that DNA structures are well-formed over large areas. All DX surfaces were annealed using Method III in the same condition as Figure 1.

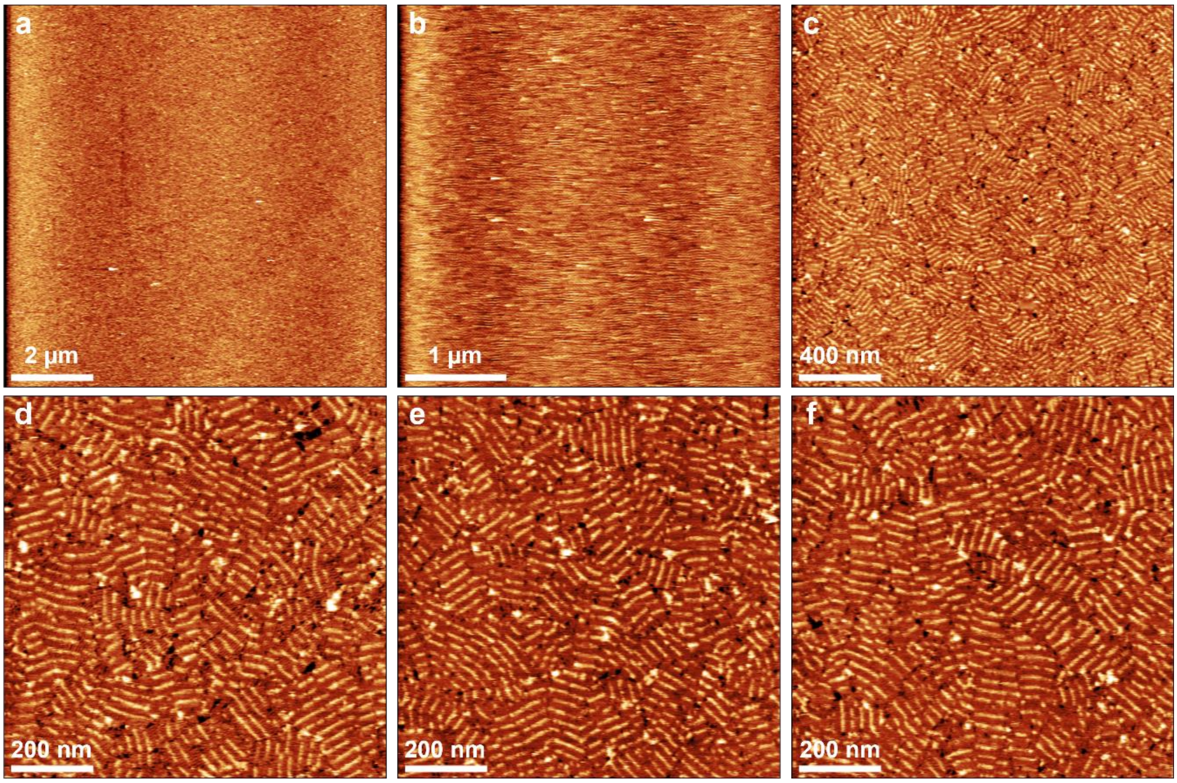

Figure S6. AFM images of 2-tile DX surface in various locations on the substrate. (a–c) AFM images of 2-tile DX surface fabricated by Method III scanned in 10 μm, 4 μm, and 2 μm. (d–f) AFM images in various 1 μm-sized areas.

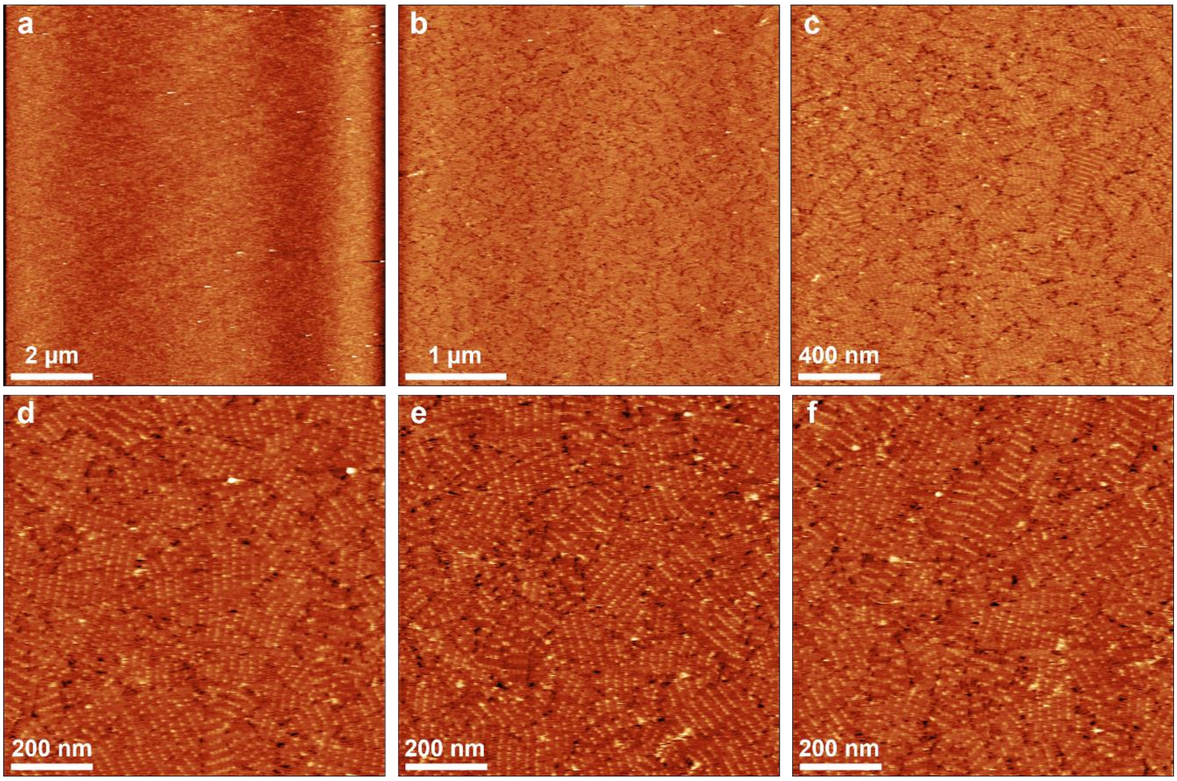

Figure S7. AFM images of 4-tile DX surface in various locations on the substrate. (a–c) AFM images of 4-tile DX surface fabricated by Method III scanned in 10 µm, 4 µm, and 2 µm. (d–f) AFM images in various 1 µm-sized areas.

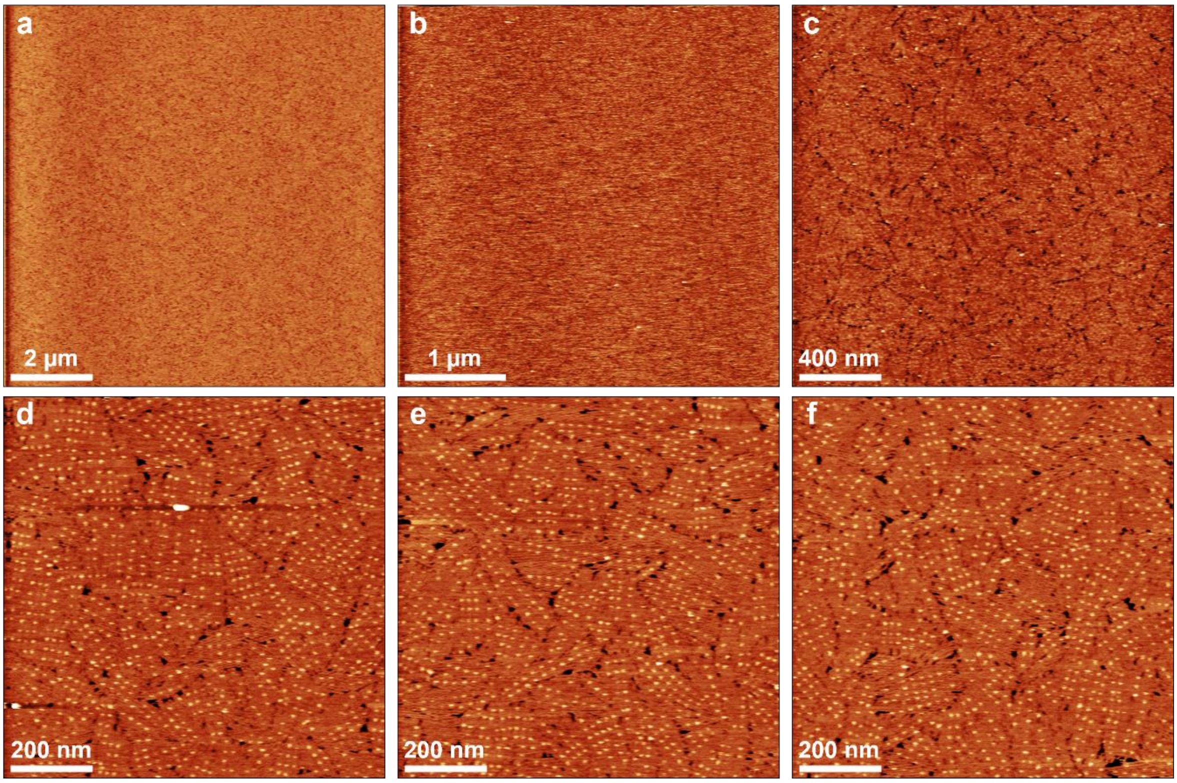

Figure S8. AFM images of 6-tile DX surface in various locations on the substrate. (a–c) AFM images of 6-tile DX surface fabricated by Method III scanned in 10 µm, 4 µm, and 2 µm. (d–f) AFM images in various 1 µm-sized areas.

Second thermal annealing condition

To investigate the influence of temperature during second thermal annealing, images of DX surfaces formed under various temperature conditions are presented below. All samples were fabricated using Method III and all processes were conducted in the same condition as Figure 2 except for the second annealing temperature.

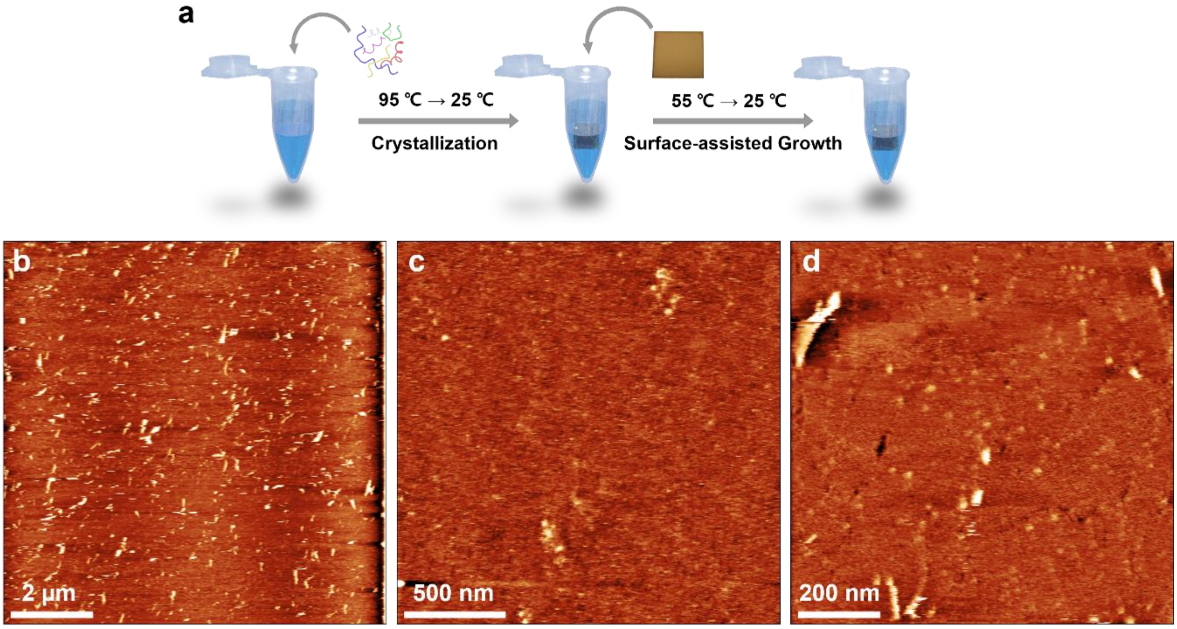

Figure S9. Analysis of the influence of temperature on surface-assisted growth at 55 °C.

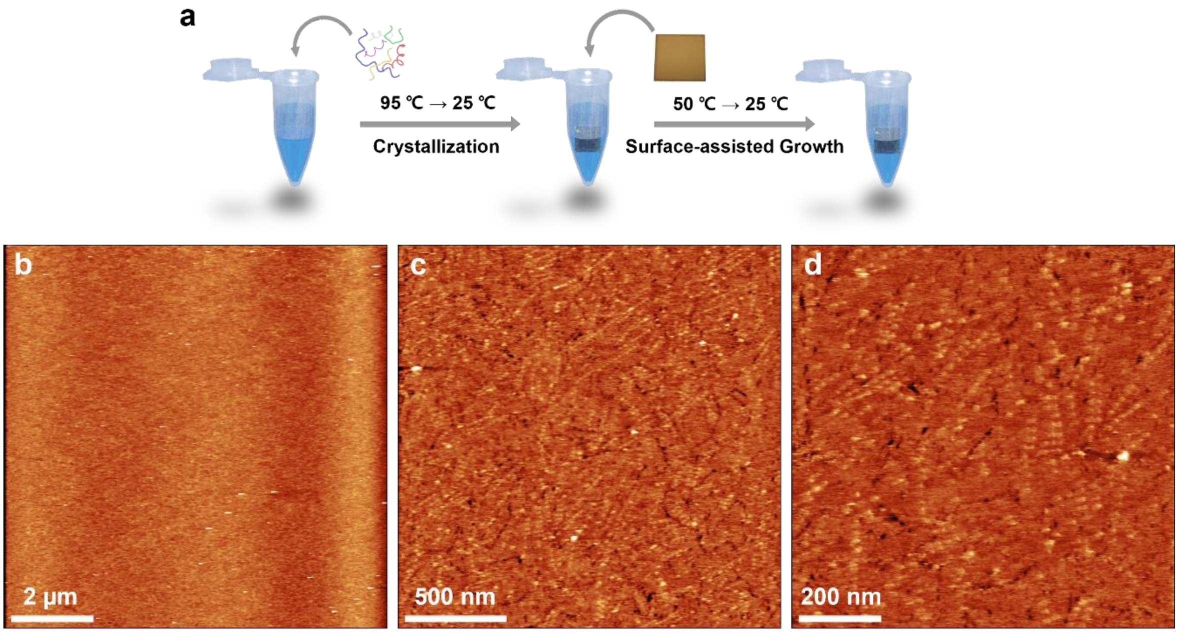

Figure S10. Analysis of the influence of temperature on surface-assisted growth at 50 °C.

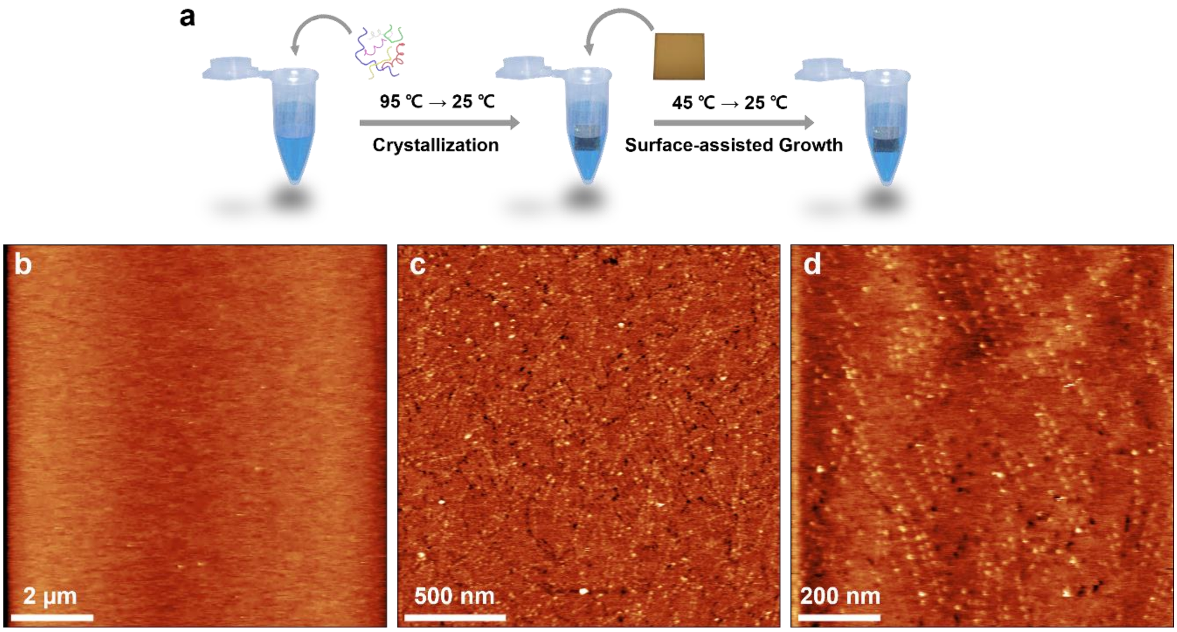

Figure S11. Analysis of the influence of temperature on surface-assisted growth at 45 °C.

AFM images of DX surfaces fabricated using Methods I and II.

AFM images of 2-tile and 4-tile DX surfaces fabricated by method I and II are presented below. Each surface was measured at 10 µm, 4 µm, and 1 µm scan size.

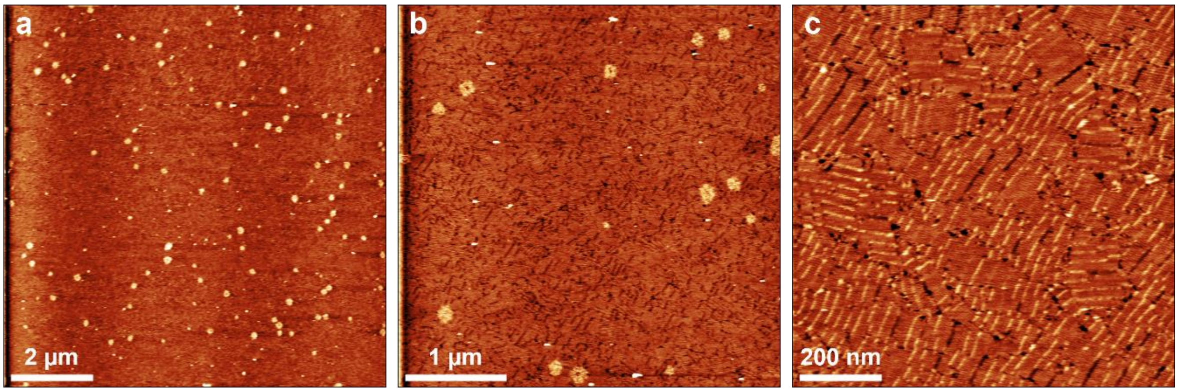

Figure S12. AFM images of 2-tile DNA nanostructure surface fabricated by Method I.

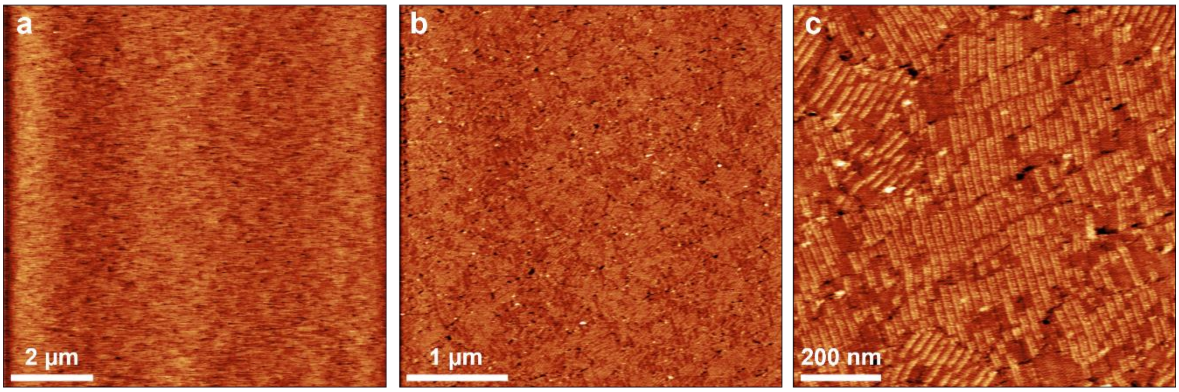

Figure S13. AFM images of 2-tile DNA nanostructure surface fabricated by Method II.

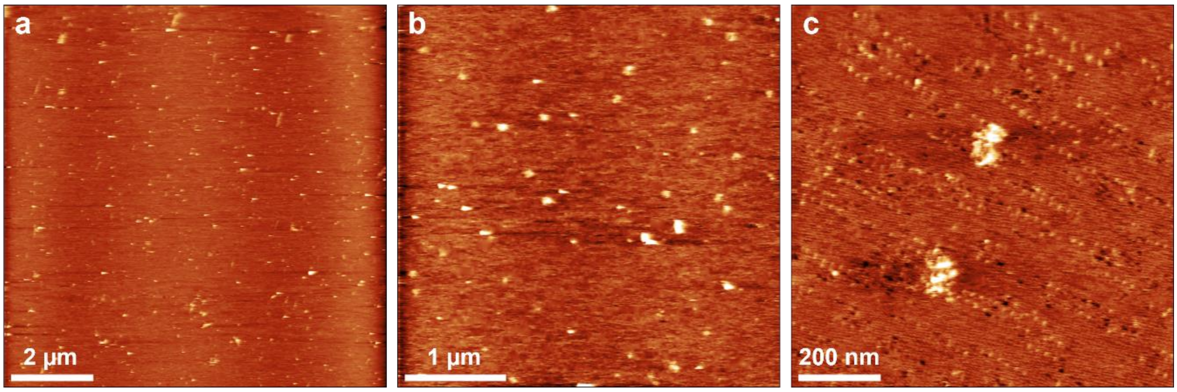

Figure S14. AFM images of 4-tile DNA nanostructure surface fabricated by Method I.

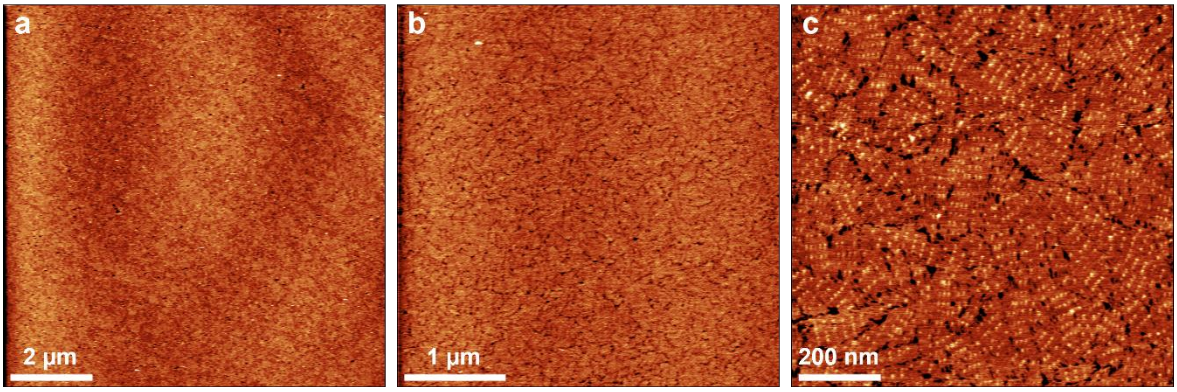

Figure S15. AFM images of 4-tile DNA nanostructure surface fabricated by Method II.

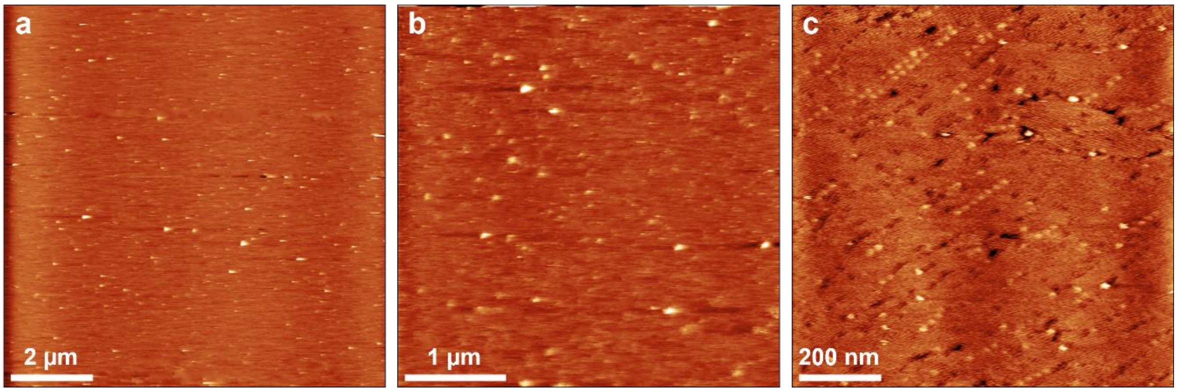

Figure S16. AFM images of 6-tile DNA nanostructure surface fabricated by Method I.

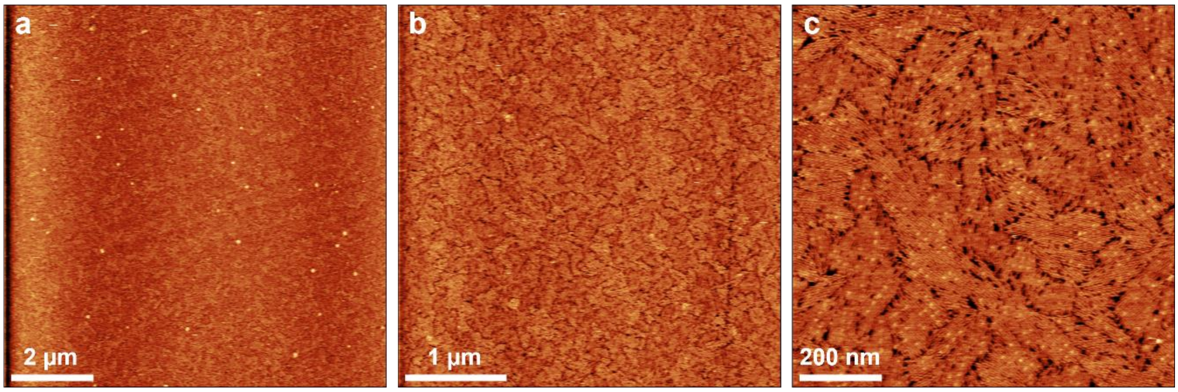

Figure S17. AFM images of 6-tile DNA nanostructure surface fabricated by Method II.

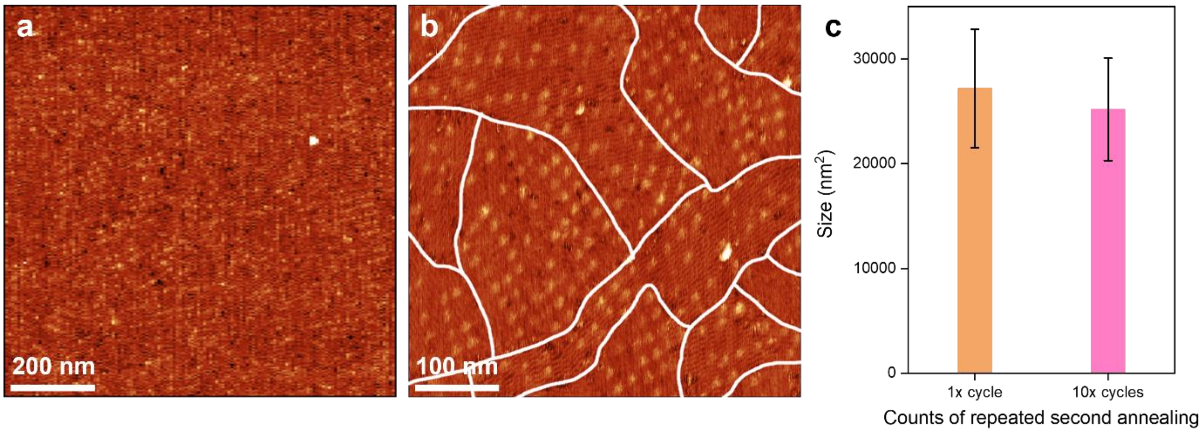

Figure S18. Analysis of the change in domain size after repeated second thermal annealing cycles. (a–b) AFM images of 6-tile domains formed on the surface after 10 cycles of second thermal annealing. (c) A bar graph representing the domain size formed after single and 10-times second thermal annealing cycles (40 °C to 25 °C). Domains were analyzed randomly in 3 different areas.

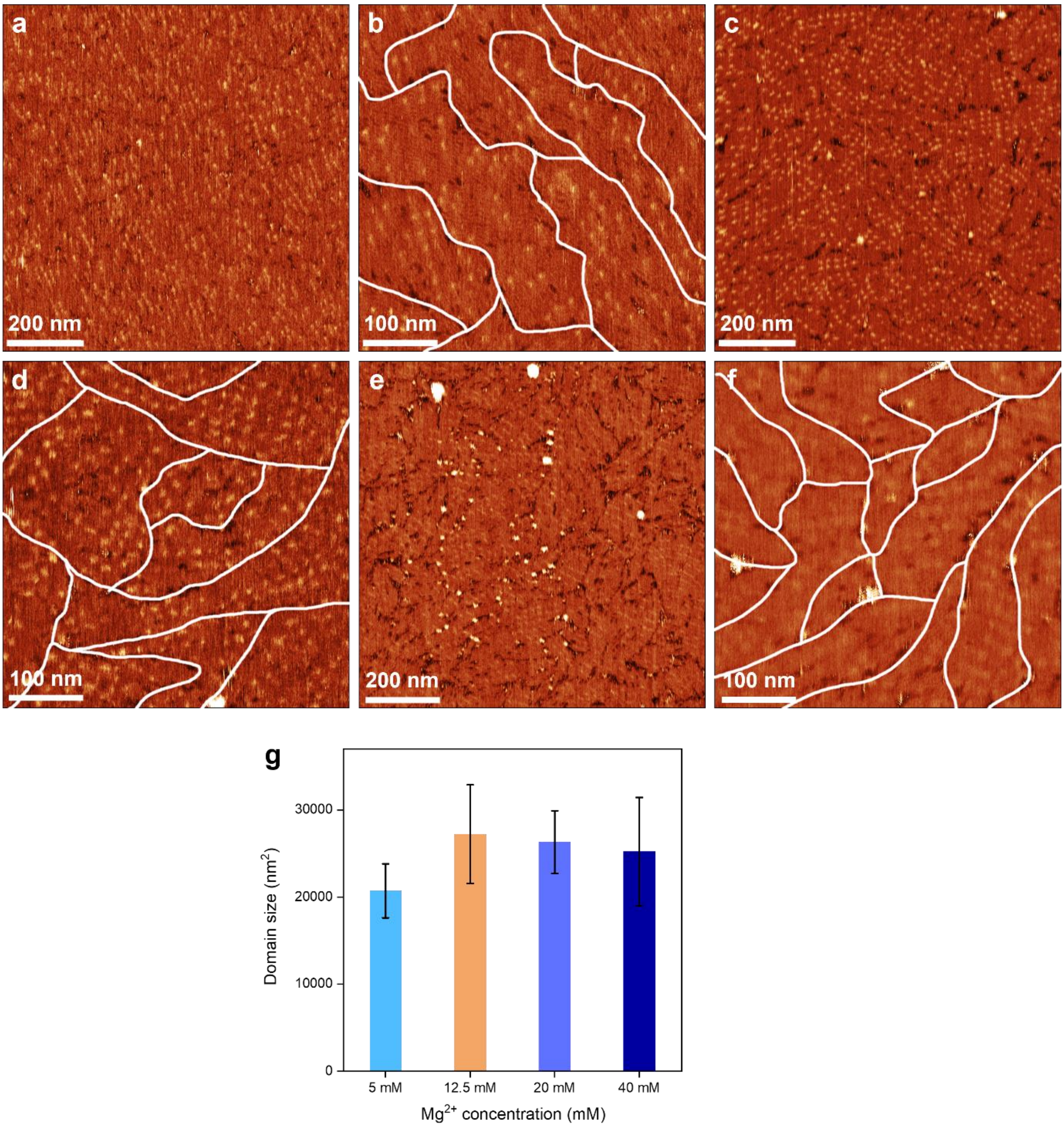

Figure S19. Analysis of surface domain size depending on Mg^{2+} concentration in second thermal annealing. Second thermal annealing was performed at different Mg^{2+} concentrations to figure out the changes in domain size due to the interaction between DX tiles and mica substrate. (a–b) AFM images of 6-tile DX surface thermally annealed in 1×TAE/5 mM Mg^{2+} buffer. (c–d) AFM images of annealed 6-tile surface in 1×TAE/20 mM Mg^{2+} buffer. (e–f) AFM images in 1×TAE/40 mM Mg^{2+} buffer. (g) A bar graph representing the domain sizes formed under each condition. All samples were analyzed using 3 images randomly scanned in different areas.

Roughness analysis.

To confirm the attachment of TBA15, the surface roughness before and after the TBA15 attachment was compared below.

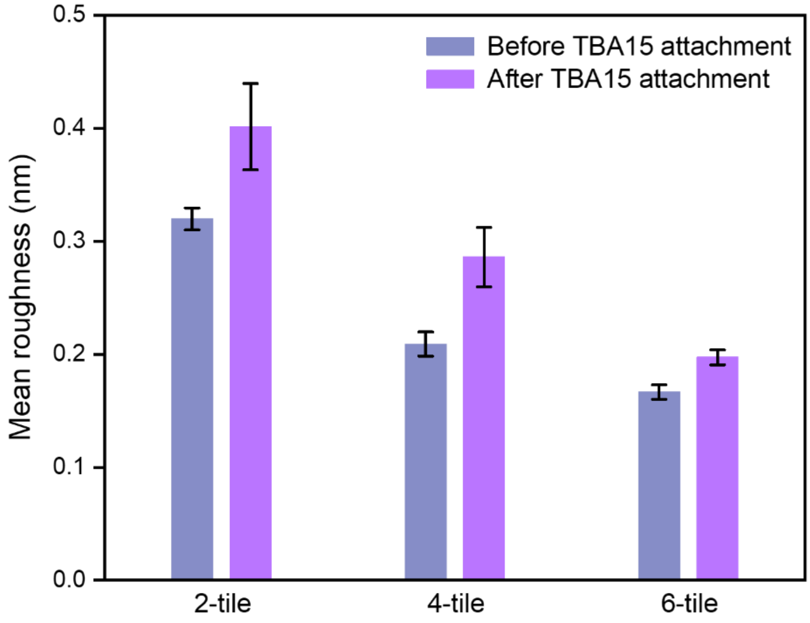

Figure S20. Changes in mean roughness of three DX surfaces due to TBA15 attachment. All DX surfaces and TBA15 attached surfaces were fabricated using Method III.

Macro-scale surface functionalization

AFM images shown below confirmed TBA15-functionalization conducted within macro-scale area on the DX surfaces.

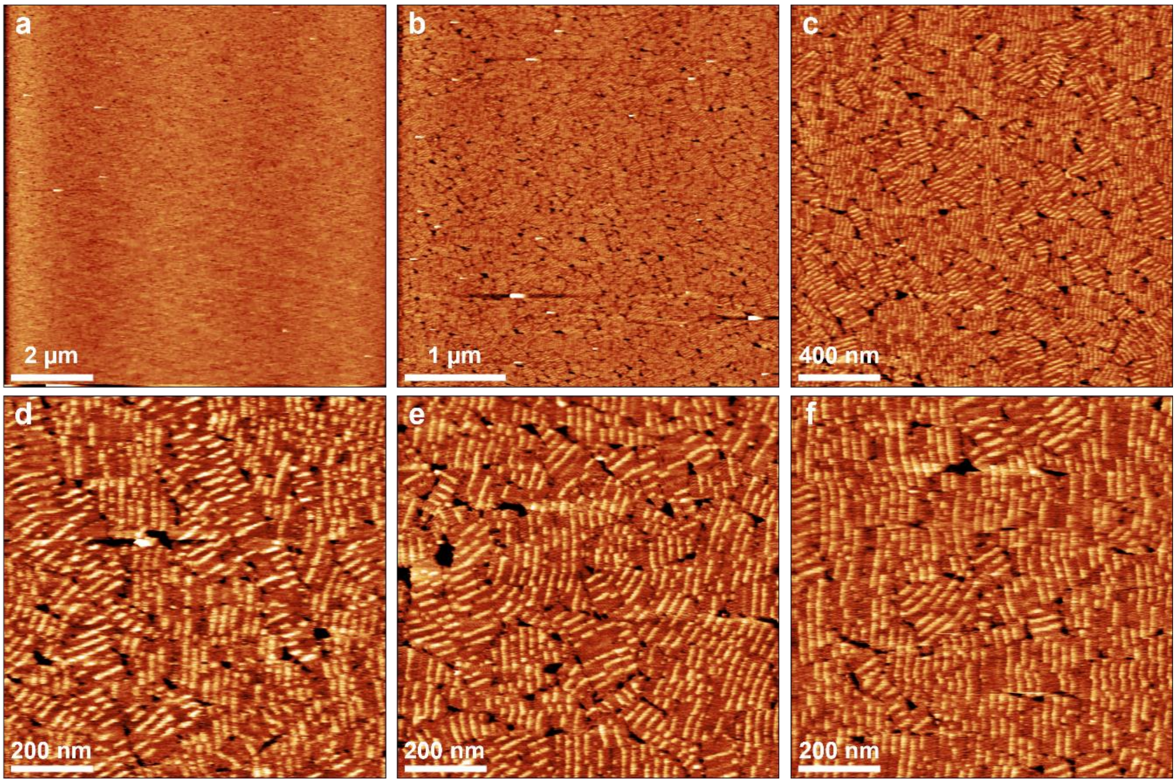

Figure S21. AFM images of 2-tile TBA15-functionalized surface in various locations on the substrate. (a–c) AFM images of 2-tile TBA15-functionalized surface scanned in 10 μm, 4 μm, and 2 μm-sized areas. (d–f) AFM images in various 1 μm-sized areas.

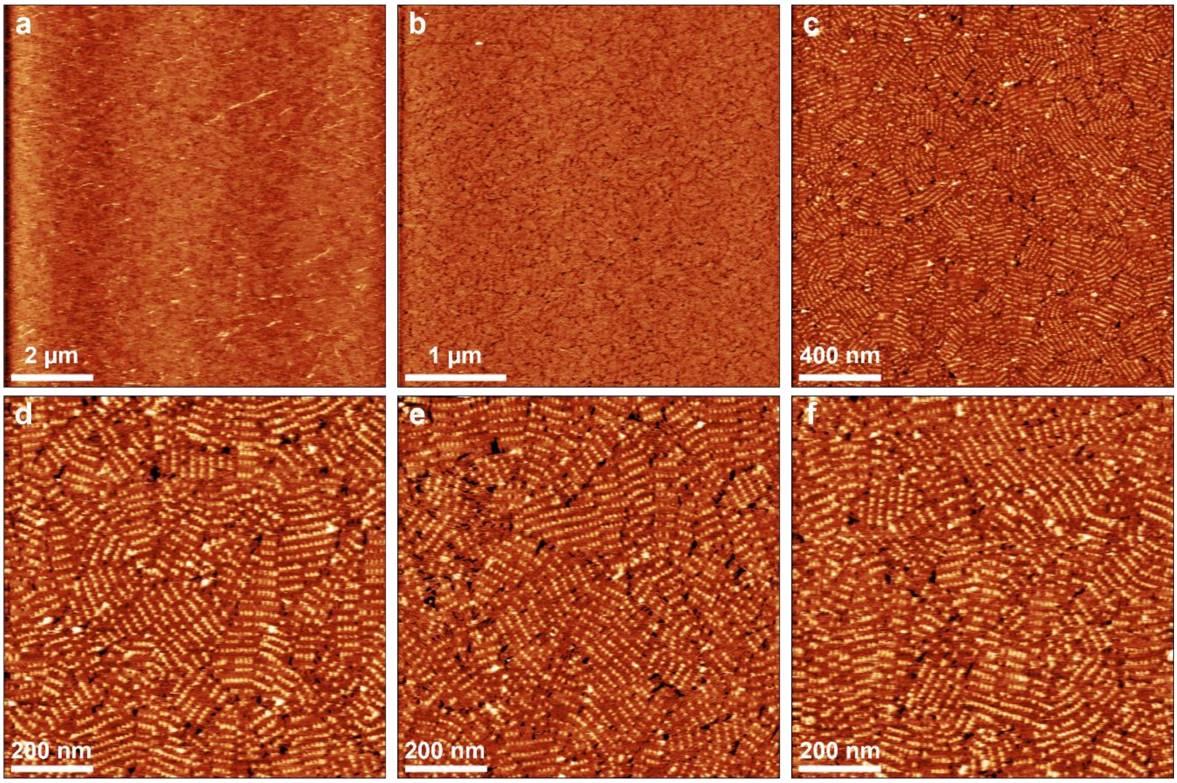

Figure S22. AFM images of 4-tile TBA15-functionalized surface in various locations on the substrate. (a–c) AFM images of 4-tile TBA15-functionalized surface scanned in 10 μm, 4 μm, and 2 μm-sized areas. (d–f) AFM images in various 1 μm-sized areas.

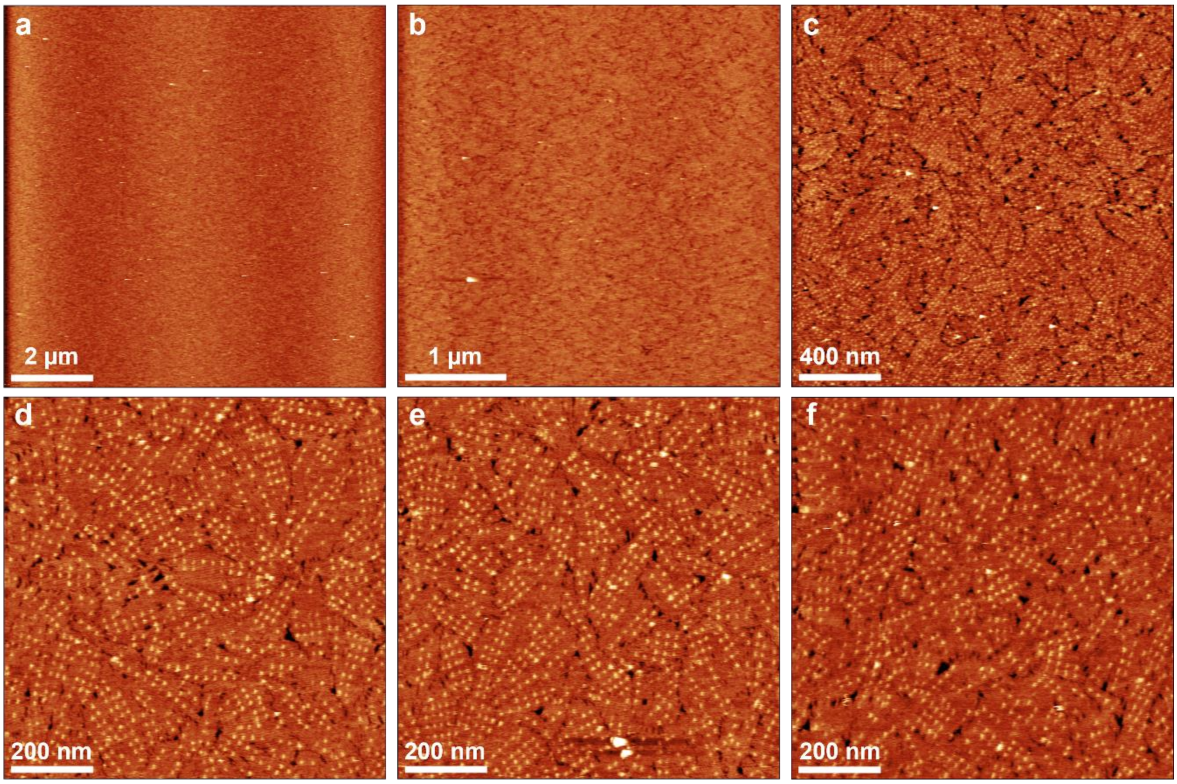

Figure S23. AFM images of 6-tile TBA15-functionalized surface in various locations on the substrate. (a–c) AFM images of 6-tile TBA15-functionalized surface scanned in 10 µm, 4 µm, and 2 µm-sized areas. (d–f) AFM images in various 1 µm-sized areas.

Chapter 3. Fluorescence Characterization

To verify if the designed TBA15 can detect thrombin and emit a fluorescence signal, changes in fluorescence signal over time (during 24 h) were investigated, and the selectivity of the TBA15-functionalized surface for thrombin was examined below.

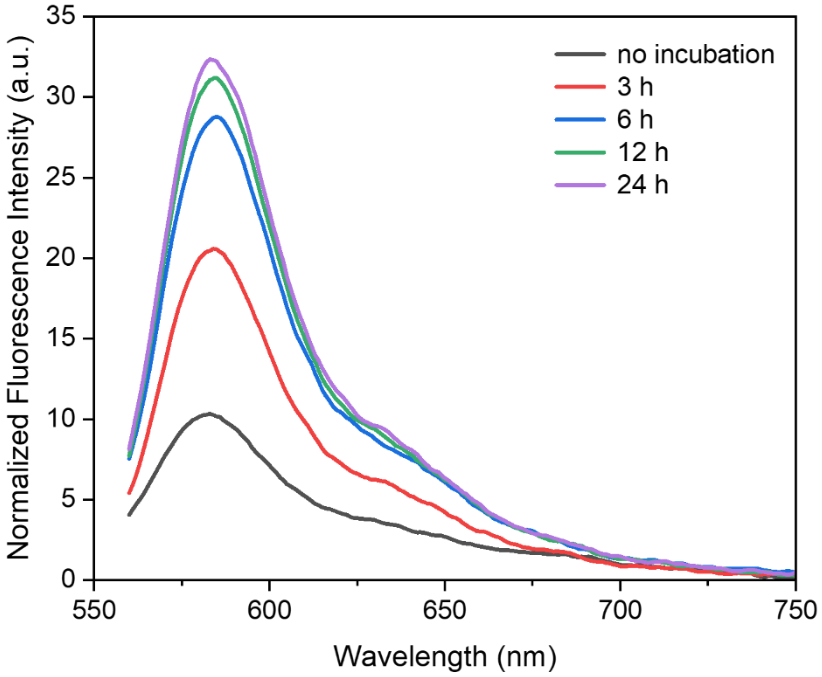

Figure S24. Fluorescence signal intensity of TBA15 over time after thrombin introduction. 500 μL of 100 nM thrombin was introduced in 500 μL of 100 nM TBA15 solution. Fluorescence intensity was measured by Photoluminescence Spectrometer (FLS 1000, Edinburgh Instruments). The excitation source was a Zenon lamp with 3.00 nm (551–553 nm) bandwidth and 1 second dwell time. Thrombin and TBA15 solution were prepared in 1×TAE/12.5 mM Mg^{2+} buffer.

Reference

(1) Hamada, S.; Murata, S. Substrate-assisted assembly of interconnected single-duplex DNA nanostructures. *Angew Chem Int Edit* **2009**, *48* (37), 6820-6823.
